# A Novel, Fast, Reliable, and Data-Driven Method for Simultaneous Single-Trial Mining and Amplitude—Latency Estimation Based on Proximity Graphs and Network Analysis

**DOI:** 10.3389/fninf.2018.00059

**Published:** 2018-11-19

**Authors:** Stavros I. Dimitriadis, Lisa Brindley, Lisa H. Evans, David E. Linden, Krish D. Singh

**Affiliations:** ^1^Cardiff University Brain Research Imaging Centre, School of Psychology, Cardiff University, Cardiff, United Kingdom; ^2^Neuroinformatics Group, Cardiff University Brain Research Imaging Centre, School of Psychology, Cardiff University, Cardiff, United Kingdom; ^3^Division of Psychological Medicine and Clinical Neurosciences, School of Medicine, Cardiff University, Cardiff, United Kingdom; ^4^School of Psychology, Cardiff University, Cardiff, United Kingdom; ^5^Neuroscience and Mental Health Research Institute, School of Medicine, Cardiff University, Cardiff, United Kingdom; ^6^MRC Centre for Neuropsychiatric Genetics and Genomics, School of Medicine, Cardiff University, Cardiff, United Kingdom; ^7^Department of Psychology, Cardiff Metropolitan University, Cardiff, United Kingdom

**Keywords:** single-trials, data-mining, proximity graphs, network analysis, amplitude, latency, reliability, signal to noise ratio (SNR)

## Abstract

Both amplitude and latency of single-trial EEG/MEG recordings provide valuable information regarding functionality of the human brain. In this article, we provided a data-driven graph and network-based framework for mining information from multi-trial event-related brain recordings. In the first part, we provide the general outline of the proposed methodological approach. In the second part, we provide a more detailed illustration, and present the obtained results on every step of the algorithmic procedure. To justify the proposed framework instead of presenting the analytic data mining and graph-based steps, we address the problem of response variability, a prerequisite to reliable estimates for both the amplitude and latency on specific N/P components linked to the nature of the stimuli. The major question addressed in this study is the selection of representative single-trials with the aim of uncovering a less noisey averaged waveform elicited from the stimuli. This graph and network-based algorithmic procedure increases the signal-to-noise (SNR) of the brain response, a key pre-processing step to reveal significant and reliable amplitude and latency at a specific time after the onset of the stimulus and with the right polarity (N or P). We demonstrated the whole approach using electroencephalography (EEG) auditory mismatch negativity (MMN) recordings from 42 young healthy controls. The method is novel, fast and data-driven succeeding first to reveal the true waveform elicited by MMN on different conditions (frequency, intensity, duration, etc.). The proposed graph-oriented algorithmic pipeline increased the SNR of the characteristic waveforms and the reliability of amplitude and latency within the adopted cohort. We also demonstrated how different EEG reference schemes (REST vs. average) can influence amplitude-latency estimation. Simulation results revealed robust amplitude-latency estimations under different SNR and amplitude-latency variations with the proposed algorithm.

## Introduction

A prerequisite for the studying of evoked potentials (EPs) is the distinction of the true brain's response due to a stimulus from the brain ongoing activity. To uncover true brain activity, a large number of single trials (STs) should be collected and averaged to reveal the brain's response waveform. The assumptions that single-trials are time-locked and contaminated by Gaussian noise of zero-mean are both oversimplified (Laskaris et al., 2004). For example, the brain state of each subject changes from time moment to time moment due to shifts of attention and the fatigue level, while habituation during the task and/or previous incidental experience with the nature of the task are significant factors that alter behavior even in short duration recordings (Laskaris and Ioannides, 2001; Laskaris et al., 2003).

Both single-trial amplitude and latency of EEG/MEG signals contain valuable information regarding brain functionality in various conditions and targeted groups. For example, increased latency variation may be associated with: ADHD (De Pascalis et al., 2008), aging (Fein and Turetsky, 1989; Fjell et al., 2009), IQ scores (Geurts et al., 2008), mild cognitive impairment (MCI; Laskaris et al., 2013), and in psychosis (Bodatsch et al., 2011).

Exploring single-trial differences between groups and/or conditions demands a proper unbiased manipulation of single-trials in order to extract reliable amplitude and latency. This is a non-trivial and demanding task for brain responses given the complexity of both brain activity and the acquired EEG/MEG recordings due to low signal-to-noise ratio (SNR) of single-trial EEG/MEG responses (Fein and Turetsky, 1989; Laskaris and Ioannides, 2001) and are usually integrated signals derived from multiple brain processes (Gevins, 1984).

Only a few exploratory studies attempted to convey information from STs. To deal with the poor SNR, different methods have been proposed in the past. The basic characteristic of previous techniques to solve the aforementioned issue based on classification and categorization of single-trials (Zouridakis et al., 1997; Geva, 1998; Lange et al., 2000). The final outcome of this procedure is the categorization of STs into homogeneous classes. Each of these classes may reflect different brain behavior like spontaneous reaction time, anticipation or reflect the variability of the regional response dynamics (Laskaris et al., 2003). Complementary, Laskaris et al., proposed a summarization of STs via Voronoi testellation procedure, minimal spanning tree, and Breadth-first graph (BFS) search procedure in order to reorder prototypical responses (Laskaris et al., 2004). The ordered prototypes reflected the variability of the single-trials while their source localization of neuromagnetic recordings with Magnetic Field Tomography (MFT) algorithm succeeded to link this variability with the related sources on different brain areas and time windows. MFT is a non-linear solution of the ill-posed biomagnetic inverse problem and it is applied independently to each single snapshot (timeslice) of either resting-state activity or single trial (or average) magnetoencephalographic (MEG) brain signal. Complementary, they very first mentioned that the ongoing activity before the onset of the stimulus is functionally coupled with the subsequent regional response (Laskaris and Ioannides, 2001; Laskaris et al., 2003). Recently, they demonstrated how the “reflex level” of spontaneous activity of various cognitive subsystems shape the brain activity during cognitive tasks stimulating the same subareas (Cole et al., 2016).

Several methods have examined STs with the goal of extracting the related amplitudes and latencies. These methods can be categorized into two groups: the ones that need an a priori template and those with no waveform constraints. A few methods need the shape of the target signal which should be defined a priori (for example, Woody, 1967; Mayhew et al., 2006). Second, only a few methods allowed the free variability of STs (for example, Pham et al., 1987; Laskaris et al., 2004), whereas others incorporate in the analytic pathway the constraint of both types of variation (for example, Jaskowski and Verleger, 1999). Third, few methods assume that the data comes from a single signal (for example, Pham et al., 1987; Jaskowski and Verleger, 1999) whereas others allow multi-trials to have their own amplitude and latency (for example, Laskaris et al., 2004; Mayhew et al., 2006; Da Pelo et al., 2018). Methodologies that analyze every trial as a unique brain response are on the right place compared to averaging across all trials. Variability of single-trials is very informative for amplitude and latency estimation and should be treated properly. Fourth, others methods provide algorithms that are susceptible to noise (Jaskowski and Verleger, 2000), whereas for others this susceptibility is reduced by incorporating basis functions. Disadvantages of the aforementioned are either the a priori selection of a template waveform derived from grand-averaged time series (Hu et al., 2011) and/or the low performance in low SNR conditions.

In optimal scenarios, principal component analysis (PCA) could be used for mining electroencephalographic and magnetoencephalographic responses (Friston et al., 1996). Alternatively, independent component analysis (ICA) could be adopted for dimensionality reduction and learning purposes of multi-trial responses (Makeig et al., 1996).

The purpose of the present paper is to demonstrate a fast, reliable, and completely data-driven methodology based on data mining, graph, and network analysis in order to reveal “true” variability of the single-trials and accurate detection of amplitude and latency linked to responses on specific stimuli. It is more than evident that single-trials are noisy even in the most optimal scenarios and experimental protocols. The motivation for the presented algorithmic steps arose after analyzing EEG STs from the famous mismatch negativity (MMN) auditory task (Näätänen et al., 2004). Single-trials were completely noisy, missing even a clear peak across trials and the multi-feature paradigm. Our analysis combined a member of proximity graph called Gabriel graph (GG) and network analysis to reveal prototypical single-trials covering the whole space of their variability and then mixed them into a combined characteristic single-trial per condition. The proposed analysis is an appropriate tool for geometrical data and vectorial pattern analysis of single-trials.

The scope of our analysis on the adopted MMN paradigm for demonstration of the methodology focused on optimizing the SNR of the selected single-trials under the objective criterion to reveal the best type of filter (IIR/FIR), its order and the degree *k* of the GG single-trial network that choose the number of selected single-trials.

## Methods

### Feature extraction

Firstly, we construct a similarity distance matrix between every pair of ST using the distance correlation estimator (Székely and Rizzo, 2009). The distance matrix called hereafter DM tabulates the distance between the temporal variability of two time series. The distance correlation is a measure of statistical dependence between two random variables or random vectors.

### Embedding in a feature space

After constructing the DM derived by the pair-wise estimation of the temporal variability of STs, we embedded the DM in a 2D feature space. Here, we adopted multidimensional scaling (MDS) a high popular dimensionality technique among neuroscientists. This approach will help us to detect and visualize the variability of STs within a common embedded feature space.

### Constructing a proximity graph on the embedded space

A proximity graph is simply a graph in which two vertices are connected by an edge if and only if the vertices satisfy particular geometric requirements. “Proximity” here means spatial distance. Many of these graphs can be formulated with respect to many metrics, but the Euclidean metric is used most frequently. Here, we used the Euclidean distance as a proper spatial distance metric.

**Figure 1:**
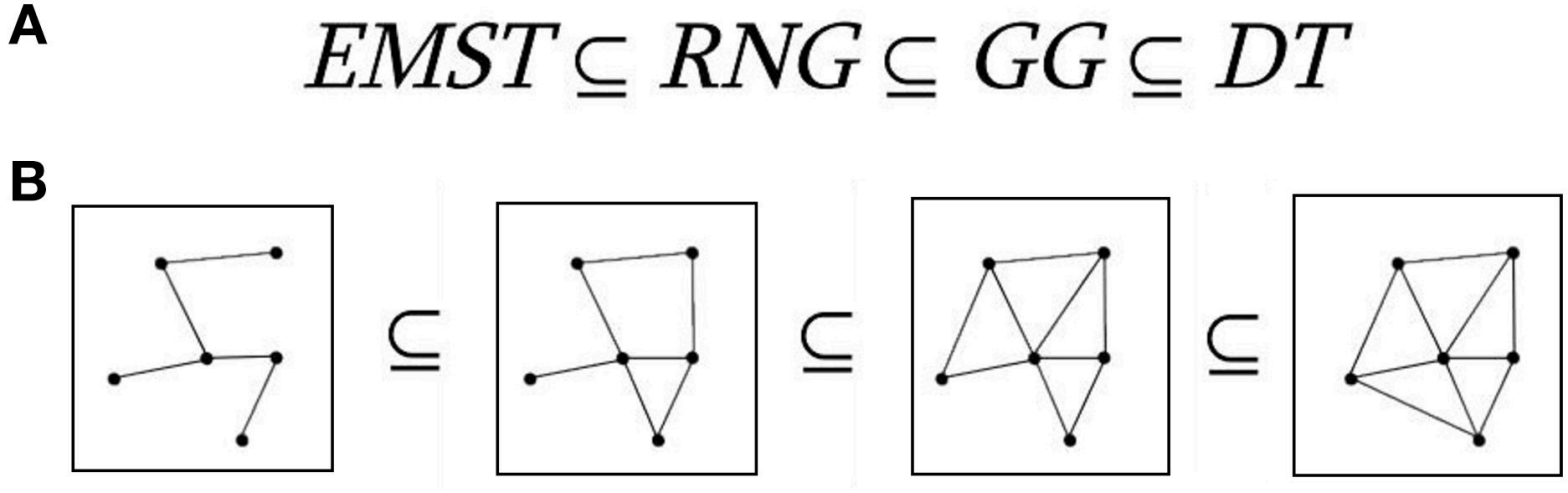
Proximity graphs. The relationship between relative neighborhood graph (RNG), Gabriel graph (GG), Euclidean minimal spanning tree (EMST), and Delaunay triangulation (DT). **(A)** Relationships between proximity graphs. **(B)** Schematic illustration of proximity graphs with five nodes.

Let L(p,q) be the intersection of the circle about p with a radius of dist(p,q) and the circle about q with a radius of dist(q,p). This is called a lune. The relative neighborhood graph RNG(V) of a set of points V, is the graph that has an edge (p,q) if and only if the intersection of L(p,q) and V is empty (Figure 1A).

Let C(p,q) be the circle centered on the point halfway between p and q, and with a radius of half the distance between p and q. The GG of a set of points V, GG(V), is the graph that has an edge (p,q) if and only if the intersection of C(p,q) and V is empty (Figure 1B).

In mathematics, the GG of a set *S* of points in the Euclidean plane expresses one notion of proximity or nearness of those points. The GG is a subgraph of the Delaunay triangulation (Matula and Sokal, 1980). Complementary, the GG contains as a subgraph the Euclidean minimum spanning tree, the RNG, and the nearest neighbor graph (Gabriel and Sokal, 1969; see Figure 1).

If we also consider the Euclidean minimum spanning tree (which is a tree that minimizes the total edge length connecting all points) and the Delauney triangulation (which maximizes the minimum angle over all triangulations of a set of points), we get the following relationship:

(1) $$\begin{array}{c} EMST\;\subseteq \;RNG\;\subseteq \;GG\;\subseteq \;DT \end{array}$$

**Figure 2:**
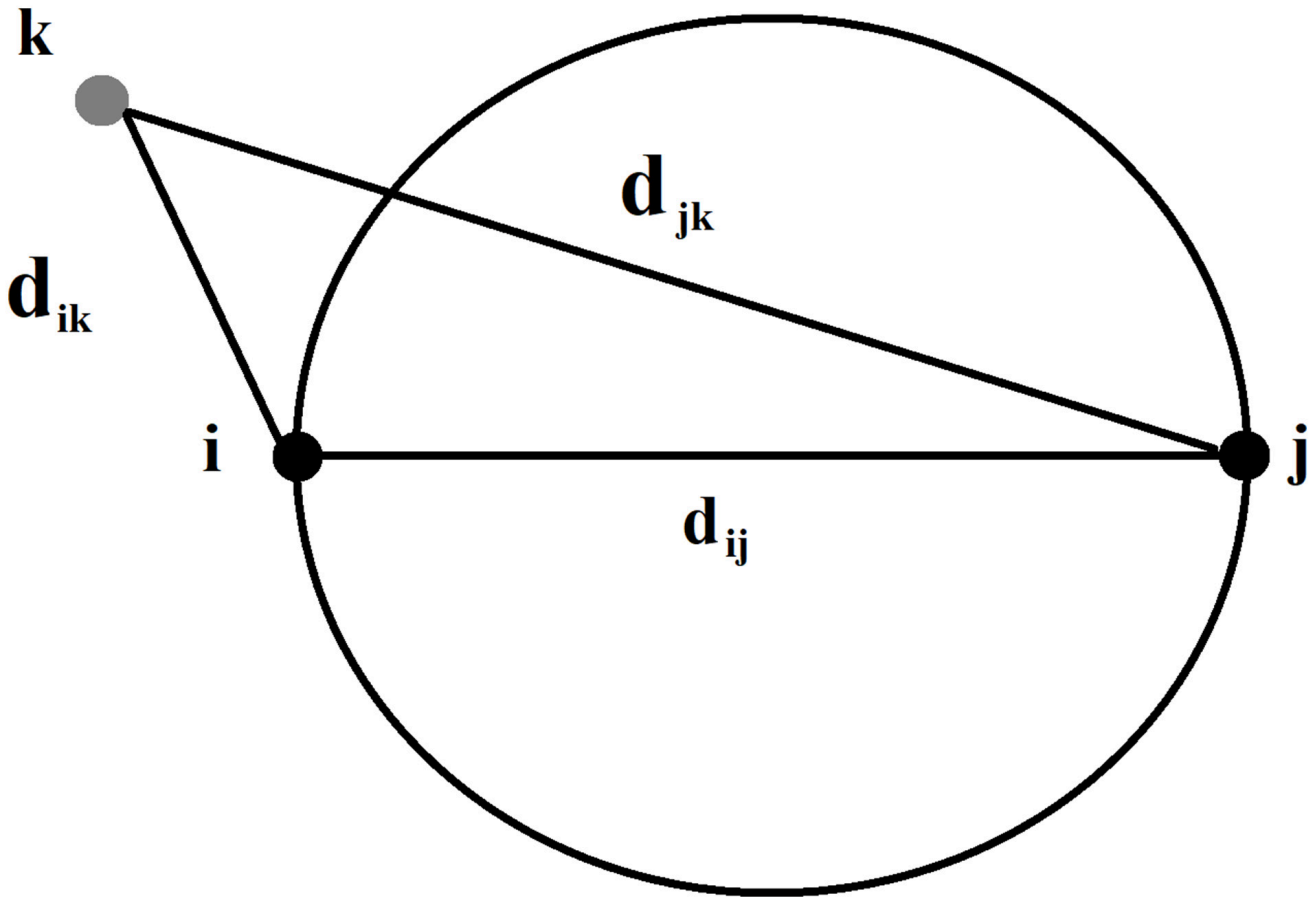
Geometric illustration of Gabriel's rule regarding the connection of two points.

Mathematically in GG, two points *i* and *j* are connected if the square of the distance between them,$d_{ij}^{2}$, is less than the sum of the squared distance between each of these points and any other point k. Under GG main rule, we connect *i* and *j* if$d_{ij}^{2}≺d_{ik}^{2}≺d_{jk}^{2}\;\left(2\right)$ for all *k points*. Schematically, we demonstrated Gabriel's rule in Figure 2.

**Figure 3:**
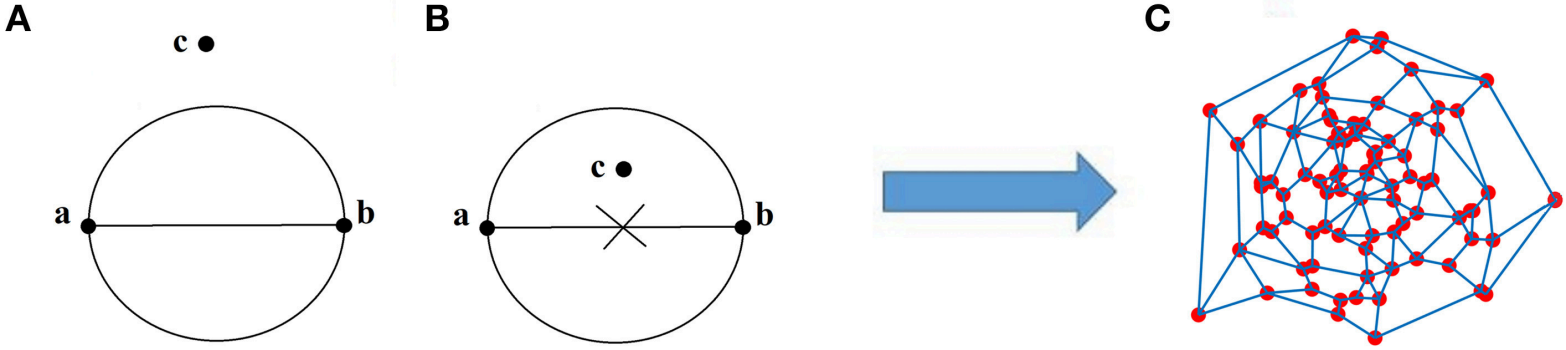
**(A)** Right, **(B)** wrong connected points in GG, and **(C)** the GG of 100 random 2D points.

Figure 3 illustrates a right (Figure 3A) and a wrong (Figure 3B) connections under the main rule of GG. Figure 3C illustrates a GG produced by 100 random points in a 2D plain. The main outcome of this approach is the construction of a GG in the 2D feature space where nodes are the single-trials. Two STs are connected if within the circle passes from their 2D coordinates no other ST is encapsulated. With this approach, GG notion demands to cover the feature space and to sample single-trial variability without over-representing. One can see GG as a denoising procedure to manipulate properly single-trials. GG captures the backbone of single-trials in the embedded space.

### Network analysis in gabriel graph (GG)

The construction of a connected GG on the 2D embedded space of single-trials opens the window to adopt well-known approachsed derived from network theory. Here, in order to detect representative prototypical STs, we used the degree of each node in GG in order to detect the hubs. The degree of a node is a trivial network metric which describes the number of direct neighbors of each node. In simple words, degree counts the number of nodes with which each node is directly connected. Here, we optimized the selection of degree k using as an optimized objective criterion the increment of SNR of grand average single-trial.

### Grand average of single trials

After selecting the prototypical STs that simultaneously capture the variability of STs, we estimated their grand-average. The selection of prototypical single-trials based on the selection of degree *k* from the GG with the aim to improve the SNR.

### Estimation of amplitude–latency

Based on the grand average signal constructed by averaging the prototypical STs, we estimated the amplitude and latency. Both amplitude and latency were extracted completely data-driven by detecting prominent peak from the whole time series as a global maxima.

### Estimating the variability of single-trials

To access the variability of STs, we estimated the global efficiency (GE) on a network level based on the subgraph defined by the nodes of GG linked to the extracted prototypical single-trials.

**Global efficiency** (*GE*) for a network *W* of *N* × *N* nodes is the inverse of the harmonic mean of the shortest path length between each pair of nodes and reflects the overall efficiency of parallel information transfer in the network (Latora and Marchiori, 2001).

(2) $$\begin{array}{r} GE=\frac{1}{N}\underset{i\in N}{\sum }\frac{\Sigma_{j\in N,j\neq i}{\left(d_{ij}\right)}^{-1}}{N-1} \end{array}$$

Here as *W*, we used the GG while *N* denotes the prototypical STs.

### MATLAB toolbox

A MATLAB toolbox will be released from the author's website, researchgate and github upon acceptance of the paper (https://github.com/stdimitr/GG_SINGLE_TRIALS_MINING/tree/master). We will demonstrate the pipeline with a few recordings which will be available to any researcher.

### Influence of reference on waveform, amplitude, and latency estimation in single-trial analysis

The influence of the reference is a critical issue for electroencephalography (EEG) and event-related potentials (ERPs) studies. It seems that brain connectivity and network analysis is more robust compared to the estimation of single power (Dimitriadis et al., 2010). A recent study proposed the infinity REST reference as an appropriate common reference system for EEG analysis (Yao, 2001; Yao et al., 2005; Qin et al., 2010; Chella et al., 2016; Huang et al., 2017). Another study compared different EEG reference systems in different simulation scenarios at both sensor and source level. They demonstrated REST infinity reference is the most preferable system across the highly used reference systems in the literature (Lei and Liao, 2017). Here, we adopted also REST reference system in comparison with the average system.

### Alternative single-trial mining algorithms

To demonstrate the superiority and the simplicity of our method compared to others, we repeated the whole analysis using PCA, singular value decomposition (SVD), and multi-linear regressor analysis (Hu et al., 2011). Both methodologies have been applied using average and REST reference.

### Simulations

We simulated the original responses using multivariate autoregressive model (MVAR) (Anderson et al., 1998) and optimized the model selection with Akaike criterion (Aikake, 1974). The simulations followed two scenarios: In the first one, both amplitude and latency parameters were the same for the peak while in the second one both amplitude-latency varies independently apart from the peak. Practically, in the first scenario, the peaks were shifted and scaled by the same amount while in the second within each trial the peak was shifted and scaled by different values.

We simulated 42 datasets (equals the number of the subjects) each consisting of 128 trials with 205 samples (400 ms). The peak varied over trials in amplitude (lognormal distributed with mean 1 and st.d. 1.2, restricted between low and high values of the empirical dataset), and latency (normally distributed with mean 0 and st.d. 150, 170, or 170 ms). The simulation was based on recordings derived from the FZ sensor at direction-left (DIR-L) condition and for deviant-minus-standard stimulus.

All simulations were performed using MVAR for the estimated waveform under three signal-to-noise (SNR) conditions (SNR = 0.5, 1, and 2), using correlated noise. Noise was simulated using an AR(5) process with coefficients estimated from baseline trials of the empirical data.

## Empirical application in an auditory mismatch negativity (MMN) protocol

The proposed methodology is demonstrated in an auditory MMN multi-feature paradigm developed by Näätänen et al. (2004). The MMN peaks at about 100–300 ms after change onset but this latency varies slightly according to the specific paradigm or the type of regularity that is violated. According to the adopted protocol, MMN is usually evoked by a change of frequency (Low-High), direction (Low-High), intensity (Low-High), duration, and gap, for both standard and deviant stimuli (Näätänen et al., 2004). Two standard tones preceded every deviant tone. Each condition was recorded in 128 trials while the protocol was designed such as to avoid any habituation of the sequence. The total number of trials was 8 × 128 for standard trials (averaged each pair of standard trials) and 2 × 128 for deviants.

We recorded a total of 42 subjects (age = 23.75 ± 1.28, 24 females) using a BIOSEMI system with 64 channels (10–20 System; Jasper, 1958). Additional electrodes were placed on the mastoid processes. The electrooculogram (EOG) was recorded from above and below the left eye [vertical (V)EOG] and from the outer canthi [horizontal (H)EOG]. The electroencephalogram (EEG; range DC-419 Hz; sampling rate 2,048 Hz) was acquired referenced to linked electrodes located midway between POz and PO3/PO4, respectively, and was re-referenced off-line to the average of the signal at the mastoids. Trials containing large EOG artifact were rejected, as were trials containing A/D saturation or baseline drift exceeding 80 μV. Prior to any further analysis, we corrected the multichannel recordings from artifacts (muscle, blinks, cardiac) using ICA with EEGLAB (Delorme and Makeig, 2004; Dimitriadis et al., 2015, 2016a,b). Data were filtered off-line (0.5–45 Hz) and down-sampled to 512 Hz, resulting in an epoch of 400 ms after the onset of the stimulus or 205 samples. DC offset was removed by subtracting from each channel each low pass filtered component using FIR filter [Roll off 0.001–0.05 Hz, butter, (IIR), 6 dB attenuation in the stop band]. Afterward, each trial was corrected with the baseline.

Our analysis focused on midline FCZ, FZ, and CZ EEG sensors. We assessed the reliability of the proposed techniques in terms of amplitude, latency, and signal power analysis.

All the subjects who participated in this study gave written informed consent. The whole study has been approved by the ethical committee in School of Psychology in Cardiff University as part of a big multi-modal study.

Our analysis on the adopted MMN paradigm for demonstration of the methodology focused on optimizing the SNR of the selected single-trials under the objective criterion to reveal the best type of filter (IIR/FIR), its order and the degree *k* applied to GG single-trial network for the selection of the representative singe-trials was the SNR (see next section).

## Results

### The proposed methodology in simple steps

**Figure 4:**
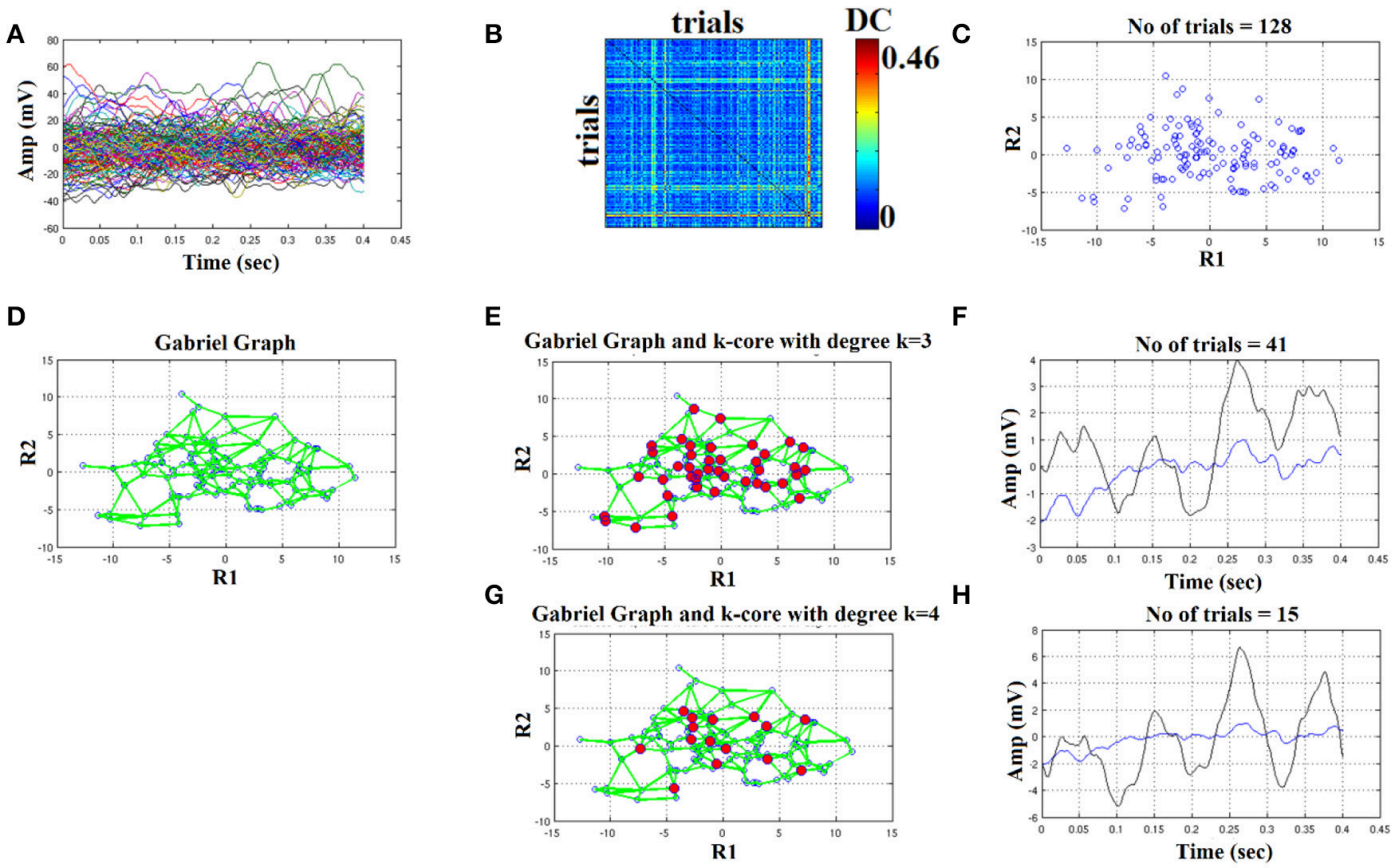
Butterworth IIR order 1: Outline of the proposed graph-based methodology (High intensity condition—deviant). **(A)** Plot of single trials from a single subject. **(B)** Similarity matrix that tabulates the pair-wise associations of single-trials with distance correlation metric. **(C)** Embedding the similarity matrix in **(B)** in a 2D space with multidimensional scaling (MDS) algorithm where each blue dot refers to a single-trial. **(D)** Construct the Gabriel graph (GG) based on the 2D positions of the single trials. Green lines represent the connections under the notion of GG. **(E)** Detection of hubs-representative single-trials based on their degree *k* in the GG. Here, we selected *k* = 3 to detect the single trials representing with red circles. **(F)** Characteristic grand-averaged single-trial derived from the averaging of the selected hubs/single trials (red circles) in **(E)**. We selected 41 signals from 128 trials. Blue waveform denotes the grand-average from the whole set of trials while the black from the selected single-trials. **(G)** Detection of hubs-representative single-trials based on their degree *k* in the GG. Here, we selected *k* = 4 [compared to 3 in **(E)**] to detect the single trials representing with red circles. **(H)** Characteristic grand-averaged single-trial derived from the averaging of the selected hubs/single trials (red circles) in **(G)**. We selected 15 signals from 128 trials. Blue waveform denotes the grand-average from the whole set of trials while the black from the selected single-trials. Amplitude and latency are estimated in the grand-averaged based on the global maxima.

We plot all the trials of a representative condition (high intensity–deviant) from a subject (Figure 4A). Afterward, we estimated with distance correlation (Székely and Rizzo, 2009), the pair-wise associations of single-trials tabulated in a similarity matrix (Figure 4B). Then, we embedded this similarity matrix in a 2D space with MDS algorithm in order to visualize the variability of single-trials (Figure 4C). Using the 2D points of the trials as an input to the GG, we constructed the GG demonstrated in Figure 4D. Green lines represent the connected trials under the notion of GG. To sample the right representative single-trials, we estimated the degree *k* of each node in GG. We used two criterion to uncover the hubs on this GG based on the degree of each node: degree *k* = 1–4. In Figures 4E,G, we demonstrated with red circles the selected trials/nodes in the GG. We selected the hubs nodes/trials based on their degree in the GG network on the assumption that these trials encapsulate the variability of the single-trials out sharing redundant information and also being on the core of variability avoiding the selection of outliers in the periphery of single-trials 2D projection.

Finally, we estimated the grand-averaged trial by averaging the selected single-trials with aforementioned network-based criterion with the objective criterion of improving the SNR of the selected single-trials. The resulting single-trial in both cases is presented in Figure 4F,H. Based on the example in Figure 4 where we used a Butterworth filter of order 1 (order since we used zero-phase filtering with filtfilt.m function of Matlab), the best result obtained with degree *k* ≥ 4 where we selected 15 trials from the 128. We detected a negative peak around 150 ms after the onset of the stimulus (**Figure 6H**). In the next section, we will demonstrate the effect of filter type, order and the selected degree in our network using as an objective criterion the improvement of SNR across the selected single-trials with our methodology.

It is important to mention here that we applied the methodology independently for standard, deviant, and deviant-minus-standard. As a promiment characteristic peak, we revealed the dominant positive for deviant, standard, and negative for deviant-minus-standard after 100 ms of the onset of the stimulus.

### The effect of FIR/IIR filter settings

**Figure 5:**
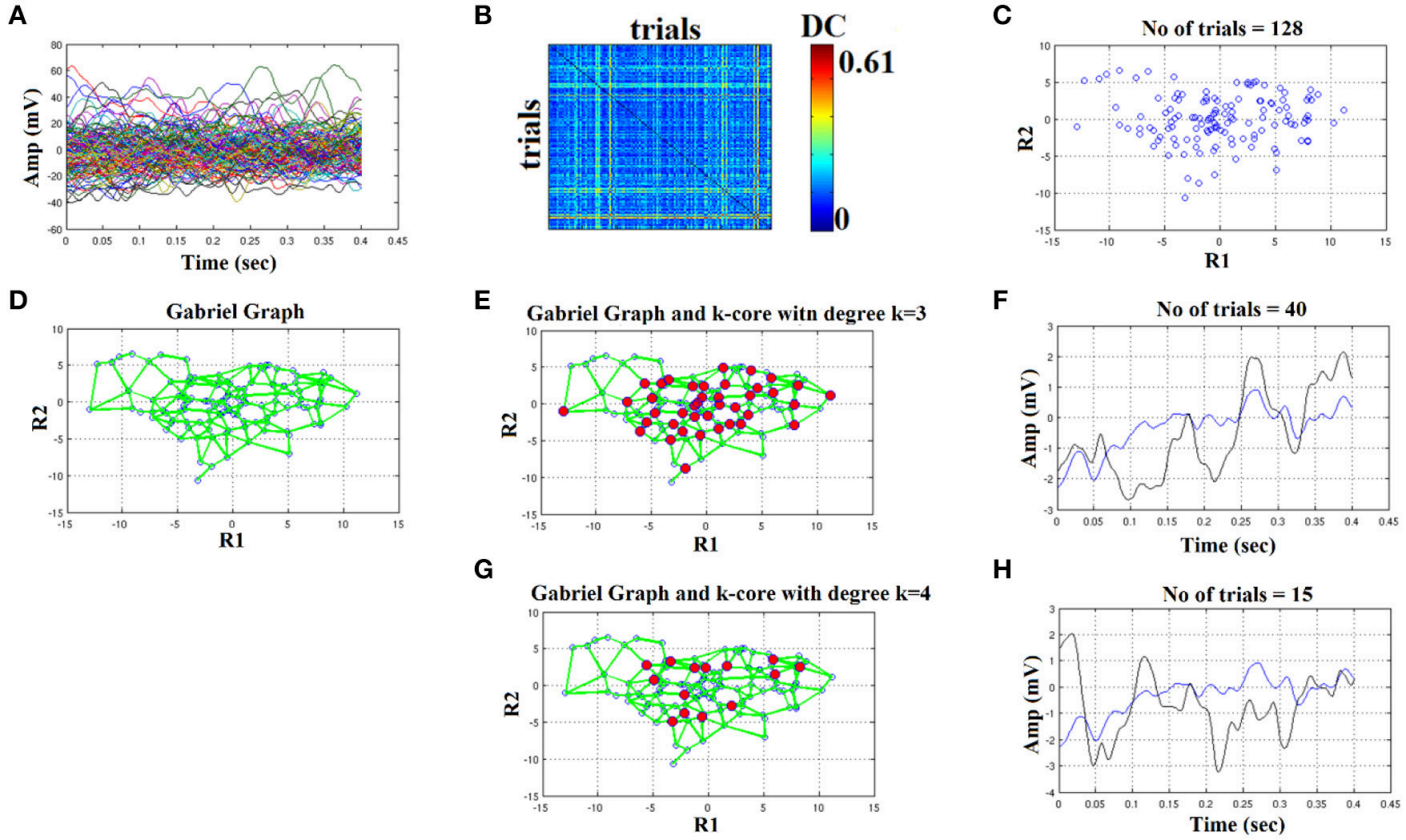
Butterworth IIR order 2: Outline of the proposed graph-based methodology (High intensity condition—deviant). **(A)** Plot of single trials from a single subject. **(B)** Similarity matrix that tabulates the pair-wise associations of single-trials with distance correlation metric. **(C)** Embedding the similarity matrix in **(B)** in a 2D space with multidimensional scaling (MDS) algorithm where every blue dot represents a single-trial. **(D)** Construct the Gabriel graph (GG) based on the 2D positions of the single trials. Green lines represent the connections under the notion of GG. **(E)** Detection of hubs-representative single-trials based on their degree *k* in the GG. Here, we selected *k* = 3 to detect the single trials representing with red circles. **(F)** Characteristic grand-averaged single-trial derived from the averaging of the selected hubs/single trials (red circles) in **(E)**. We selected 40 signals from 128 trials. Blue waveform denotes the grand-average from the whole set of trials while the black from the selected single-trials. **(G)** Detection of hubs-representative single-trials based on their degree *k* in the GG. Here, we selected *k* = 4 [compared to 3 in **(E)**] to detect the single trials representing with red circles. **(H)** Characteristic grand-averaged single-trial derived from the averaging of the selected hubs/single trials (red circles) in **(G)**. We selected 25 signals from 128 trials. Blue waveform denotes the grand-average from the whole set of trials while the black from the selected single-trials. Amplitude and latency are estimated in the grand-averaged based on the global maxima.

**Figure 6:**
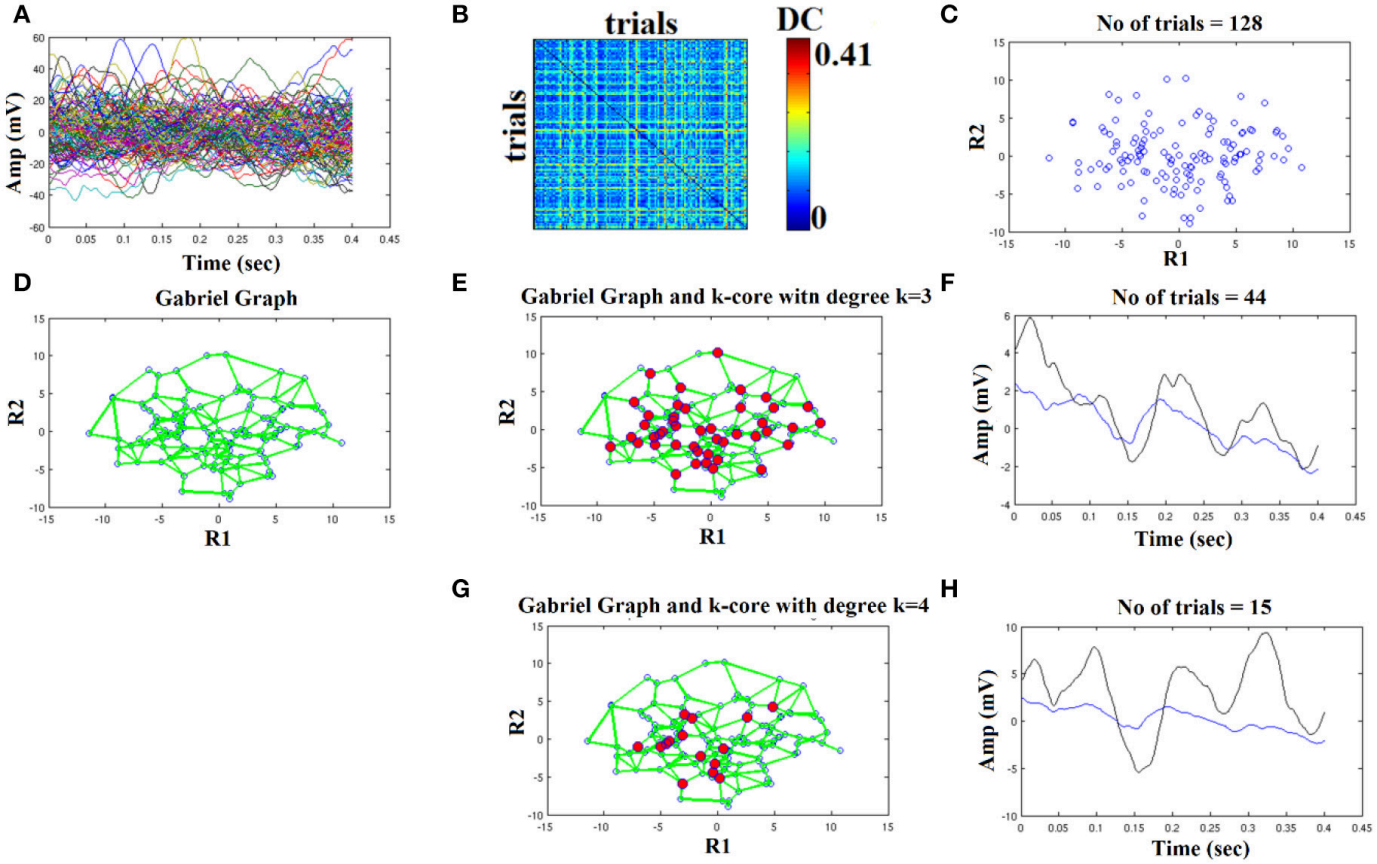
FIR order 2: Outline of the proposed graph-based methodology (High intensity condition—deviant). **(A)** Plot of single trials from a single subject. **(B)** Similarity matrix that tabulates the pair-wise associations of single-trials with distance correlation metric. **(C)** Embedding the similarity matrix in **(B)** in a 2D space with multidimensional scaling (MDS) algorithm where every blue dot represents a single-trial. **(D)** Construct the Gabriel graph (GG) based on the 2D positions of the single trials. Green lines represent the connections under the notion of GG. **(E)** Detection of hubs-representative single-trials based on their degree *k* in the GG. Here, we selected *k* = 3 to detect the single trials representing with red circles. **(F)** Characteristic grand-averaged single-trial derived from the averaging of the selected hubs/single trials (red circles) in **(E)**. We selected 44 signals from 128 trials. Blue waveform denotes the grand-average from the whole set of trials while the black from the selected single-trials. **(G)** Detection of hubs-representative single-trials based on their degree *k* in the GG. Here, we selected *k* = 4 [compared to 3 in **(E)**] to detect the single trials representing with red circles. **(H)** Characteristic grand-averaged single-trial derived from the averaging of the selected hubs/single trials (red circles) in **(G)**. We selected 15 signals from 128 trials. Blue waveform denotes the grand-average from the whole set of trials while the black from the selected single-trials. Amplitude and latency are estimated in the grand-averaged based on the global maxima.

In Figures 4–**6**, we demonstrated the steps of the proposed fast, reliable, and data-driven methodology under a graph-based framework. We revealed that both the type of the filter (FIR/IIR) and its order can alter the characteristic waveform for each condition and subject.

We used the eegfilt matlab function a provided in EEGLAB for FIR filtering of single-trials (Delorme and Makeig, 2004) and the butter MATLAB function for IIR filtering. We used a zero-phase filter in both cases applied on the concatenated trials separately for each stimulus (standard or deviant) and for each subject. The effect of filter with Butterworth bandpass filter can be seen on the representative time series in Figure 4H (order 1) vs. Figure 5H (order 2). Order of one gave the best results for Butterworth bandpass IIR filter. In contrary, the best result for FIR filter using eegfilt function was obtained with order 2 (Figure 6H). In Figure 4H, the characteristic negative was detected around 100 ms after the onset of the stimulus while in Figure 6H, the negative peak located 150 ms after the onset of the stimulus. The effect of type and order of filter was demonstrated in high intensity condition for deviant stimulus from a single subject. Our analysis on the adopted MMN paradigm for demonstration of the methodology revealed as the best option based on SNR for filtering the FIR using eegfilt function and with order 2. The main objective criterion to reveal the best type of filter (IIR/FIR), order and degree *k* for the selection of the representative singe-trials was the SNR (see next section).

### Improvement of signal-to-noise ratio with the proposed methodology

We evaluated the selection of the filter type, its order and the degree *k* in GG for the selection of single-trials independently for each condition, standard/deviant, subject and recording EEG sensors (FZ, FCZ, CZ). The parameters were filter type (FIR/IIR), order (1,2,3), and degree *k* (1–4). Finally, we estimated the SNR from the selected single-trials via the proposed methodology, adopting a formula previously proposed (Laskaris et al., 2004). We scored each of the 2 × 3 × 4 = 24 different sets of parameters across conditions (8 deviant and 8 standard stimuli = 16) and recording EEG sites (three locations) the number of times where the SNR was maximum across the 24 sets. Formula (3) describes the objective criterion for the selection of best settings for each subject across the 24 different combinations. Our results demonstrated clearly a maximization of SNR for every subject with FIR filter of order 2 and with most of the cases (39 out of 42) with degree *k* ≥ 4.

(3) $$Score=\frac{\sum_{filter=1}^{2}\sum_{order=1}^{3}\sum_{\deg ree=1}^{4}\arg\max\;SNR(16\;stimuli,\;3\;recording\;sites)}{16\;stimuli\;x\;3\;stimuli}$$

The group-averaged score was 96.34 with standard deviation 2.31 with the best choice for the FIR filter (42 out of 42 subjects), order 2 (42 out of 42 subjects), and *k* = 4 (39 out of 42 subjects and 3 with *k* = 3). Table 1 summarizes the group-averaged SNR from each stimulus and EEG sensor location for the standard stimuli. We presented results from FZ location where the majority of group-differences in terms of amplitude, latency, signal power, and variability were more pronounced compared to FCZ and CZ.

**Table 1:** Group-averaged SNR for each condition across the three selected EEG sensors for standard stimuli.

	**Dir-L**	**Dir-R**	**Freq-Hi**	**Freq-Low**	**Int-Low**	**Int-High**	**Duration**	**Gap**
FZ	6.14 ± 1.01	5.62 ± 0.78	6.23 ± 1.14	6.47 ± 1.07	6.68 ± 1.34	6.39 ± 0.77	6.71 ± 0.91	6.84 ± 1.43
FCZ	5.94 ± 1.13	5.67 ± 0.92	5.90 ± 0.98	6.07 ± 1.19	6.07 ± 1.31	5.78 ± 1.31	6.39 ± 1.14	6.16 ± 0.91
CZ	5.47 ± 0.87	5.44 ± 1.12	5.67 ± 1.10	5.61 ± 0.87	5.76 ± 1.45	5.45 ± 1.12	5.76 ± 1.13	5.93 ± 1.14

Results of SNR for the grand-averaged signal was <1 and one can see in Figures 3–6, it is a bad strategy to estimate a peak for this noisy averaged trial (blue line in Figures 4–6F,H).

### Reliability of amplitude, latency, and signal power

We assessed the reliability of amplitude, latency and signal power estimates for each MMN feature, EEG sensors and for standard, deviant, and deviant-minus-standard with the coefficient of variation (CV). The CV was estimated as follow:

(4) $$\begin{array}{r} CV=\frac{group\;mean\left(amplitude,\;latency\right)}{group\;std\left(amplitude,\;latency\right)} \end{array}$$

### Amplitude and latencies

Tables 2–4 demonstrated the group mean amplitude for standard, deviant, and deviant – standard for each condition of the MMN experimental protocol and for the three EEG sensors. We estimated the CV (Formula 4) across the cohort for every MMN feature for standard, deviant, and deviant – standard and for FZ (Table 2), FCZ (Table 3), and CZ (Table 4) EEG sensors. It is obvious that CV of the amplitude was higher for FZ EEG sensor.

**Table 2:** FZ EEG sensor: Group-averaged amplitude for each condition and for standard, deviants, and their difference (deviant-minus-standard).

	**Dir-R**	**Dir-L**	**Freq-Hi**	**Freq-Low**	**Int-Hi**	**Int-Low**	**Duration**	**Gap**
Std	3.9 ± 1.3(3)	4.1 ± 1.2(3.78)	4.0 ± 1.2(3.5)	4.3 ± 1.4(3.1)	4.1 ± 0.9(4.2)	3.7 ± 1.5(2.4)	2.3 ± 0.7(3.2)	2.4 ± 0.7(3.3)
Dev	−3.5 ± 1.1(3.2)	−5.3 ± 1.2(4.4)	−3.5 ± 1.1(3.2)	−3.9 ± 1.2(3.4)	−3.9 ± 1.4(3.7)	−4.1 ± 1.2(3.6)	−2.4 ± 0.6(4)	−2.5 ± 0.5(5)
Dev-Std	−4.7 ± 1.2(3.89)	−6.4 ± 1.5(4.12)	−4.7 ± 1.4(3.3)	−5.4 ± 1.4(3.8)	−4.2 ± 1.2(3.5)	−4.3 ± 1.4(3.2)	−3.2 ± 0.5(6.4)	−3.1 ± 0.6(5.1)

*Within the brackets we report the coefficient of variation (CV)*.

**Table 3:** FCZ EEG sensor: Group-averaged amplitude for each condition and for standard, deviants, and their difference (deviant-minus-standard).

	**Dir-R**	**Dir-L**	**Freq-Hi**	**Freq-Low**	**Int-Hi**	**Int-Low**	**Duration**	**Gap**
Std	3.5 ± 1.6(2.2)	3.8 ± 1.3(3.9)	3.7 ± 1.3(2.8)	3.6 ± 1.3(2.7)	3.8 ± 1.1(3.4)	3.6 ± 1.3(2.7)	2.4 ± 0.7(3.4)	2.2 ± 0.6(3.75)
Dev	−3.2 ± 1.6(2)	−3.9 ± 1.3(3)	−3.6 ± 1.3(2.8)	−3.7 ± 1.3(2.8)	−2.5 ± 1.1(2.2)	−3.3 ± 1.3(2.6)	−2.5 ± 0.6(4.25)	−2.6 ± 0.7(3.6)
Dev-Std	−4.7 ± 1.6(2.9)	−3.7 ± 1.6(2.3)	−4.1 ± 1.4(2.9)	−3.9 ± 1.4(2.8)	−4.1 ± 1.3(3.2)	−4.0 ± 1.4(2.8)	−3.3 ± 0.9(3.5)	−3.2 ± 0.8(4)

*Within the brackets we report the coefficient of variation (CV)*.

**Table 4:** CZ EEG sensor: Group-averaged amplitude for each condition and for standard, deviants, and their difference (deviant-minus-standard).

	**Dir-R**	**Dir-L**	**Freq-Hi**	**Freq-Low**	**Int-Hi**	**Int-Low**	**Duration**	**Gap**
Std	3.2 ± 1.3(2.5)	2.4 ± 1.0(2.4)	2.3 ± 1.0(2.3)	2.3 ± 0.9(2.5)	2.4 ± 0.8(3)	1.9 ± 0.7(2.4)	1.9 ± 0.6(3.1)	2.5 ± 0.7(3.4)
Dev	−2.2 ± 1.0(2.2)	−2.3 ± 1.1(2.89)	−2.3 ± 0.9(2.6)	−2.2 ± 0.8(2.7)	−2.5 ± 1.0(2.5)	−2.0 ± 0.8(2.5)	−2.1 ± 0.7(2.7)	−2.6 ± 0.6(4.3)
Dev-Std	−3.5 ± 1.2(2.9)	−3.4 ± 1.3(4.12)	−3.1 ± 1.1(2.8)	−3.3 ± 1.4(2.4)	−3.2 ± 1.3(2.5)	−2.4 ± 1.1(2.2)	−2.3 ± 0.9(2.6)	−3.3 ± 0.7(4.6)

*Within the brackets we report the coefficient of variation (CV)*.

Tables 5–7 demonstrated the group mean latencies for standard, deviant, and deviant – standard for each condition of the MMN experimental protocol and for the three EEG sensors. We estimated the CV across the cohort for every MMN feature for standard, deviant, and deviant – standard and for FZ (Table 5), FCZ (Table 6), and CZ (Table 7) EEG sensors. It is obvious that CV of the latency was higher for FZ EEG sensor.

**Table 5:** FZ EEG sensor: Group-averaged latency for each condition and for standard, deviants, and their difference (deviant-minus-standard).

	**Dir-R**	**Dir-L**	**Freq-Hi**	**Freq-Low**	**Int-Hi**	**Int-Low**	**Duration**	**Gap**
Std	0.18 ± 0.03 (6)	0.20 ± 0.03 (6.66)	0.18 ± 0.02 (9)	0.21 ± 0.03 (7)	0.20 ± 0.03 (6.66)	0.17 ± 0.03 (5.66)	0.19 ± 0.03 (6.3)	0.17 ± 0.03 (5.6)
Dev	0.19 ± 0.03 (6.3)	0.18 ± 0.03 (6)	0.17 ± 0.03 (5.66)	0.18 ± 0.02 (9)	0.19 ± 0.02 (9.5)	0.16 ± 0.03 (5.3)	0.18 ± 0.03 (6)	0.17 ± 0.03 (5.6)
Dev-Std	0.17 ± 0.02 (8.5)	0.16 ± 0.02 (8)	0.16 ± 0.02 (8)	0.17 ± 0.03 (5.6)	0.17 ± 0.03 (5.66)	0.16 ± 0.02 (8)	0.16 ± 0.03 (5.3)	0.16 ± 0.02 (8)

*Within the brackets we report the coefficient of variation (CV)*.

**Table 6:** FCZ EEG sensor: Group-averaged latency for each condition and for standard, deviants, and their difference (deviant-minus-standard).

	**Dir-R**	**Dir-L**	**Freq-Hi**	**Freq-Low**	**Int-Hi**	**Int-Low**	**Duration**	**Gap**
Std	0.20 ± 0.03 (6.6)	0.17 ± 0.03 (5.6)	0.18 ± 0.03 (5.6)	0.17 ± 0.03 (5.6)	0.17 ± 0.03 (5.6)	0.16 ± 0.03 (5.3)	0.18 ± 0.03 (6)	0.19 ± 0.03 (6.3)
Dev	0.20 ± 0.02 (10)	0.19 ± 0.03 (6.3)	0.19 ± 0.03 (6.3)	0.16 ± 0.02 (8)	0.18 ± 0.03 (6)	0.19 ± 0.03 (6.3)	0.22 ± 0.03 (7.3)	0.22 ± 0.03 (7.3)
Dev-Std	0.19 ± 0.02 (9.5)	0.20 ± 0.02 (10)	0.21 ± 0.02 (10.5)	0.19 ± 0.03 (6.3)	0.16 ± 0.03 (5.3)	0.17 ± 0.02 (8.5)	0.17 ± 0.03 (5.6)	0.21 ± 0.03 (7)

*Within the brackets we report the coefficient of variation (CV)*.

**Table 7:** CZ EEG sensor: Group-averaged latency for each condition and for standard, deviants, and their difference (deviant-minus-standard).

	**Dir-R**	**Dir-L**	**Freq-Hi**	**Freq-Low**	**Int-Hi**	**Int-Low**	**Duration**	**Gap**
Std	0.17 ± 0.02 (8.5)	0.18 ± 0.03 (6)	0.17 ± 0.02 (8.5)	0.17 ± 0.03 (6.3)	0.17 ± 0.03 (6.3)	0.17 ± 0.02 (8.5)	0.18 ± 0.03 (6)	0.19 ± 0.03 (6.3)
Dev	0.18 ± 0.03 (6)	0.21 ± 0.03 (7)	0.19 ± 0.03 (6.3)	0.16 ± 0.02 (8)	0.17 ± 0.02 (8.5)	0.18 ± 0.03 (6)	0.21 ± 0.03 (7)	0.18 ± 0.03 (6)
Dev-Std	0.19 ± 0.02 (9.5)	0.18 ± 0.02 (9)	0.18 ± 0.02 (9)	0.18 ± 0.03 (6)	0.16 ± 0.03 (5.3)	0.19 ± 0.02 (9.5)	0.20 ± 0.03 (6.6)	0.21 ± 0.02 (10.5)

*Within the brackets we report the coefficient of variation (CV)*.

We repeated the whole analysis by selecting a subset of single-trials from each condition and subject starting from the first 20% of the trials per condition till 100% with a step of 5% in order to explore how the number of trials affect amplitude/latency estimations. We revealed that the CV of amplitude/latency reached high values close to the ones tabulated in Tables 2–7 when the number of trials ranged between [85–95%] of the total amount of STs. The aforementioned results underline the importance of detect significant true amplitude/latency estimations in MMN paradigm.

### Signal power

**Figure 7:**
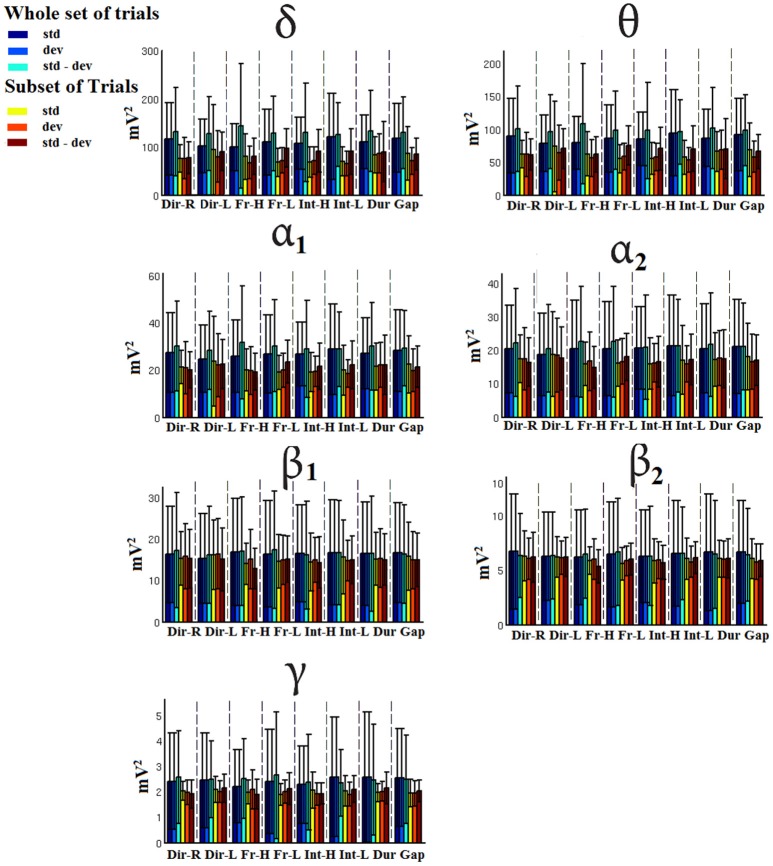
FZ-Group-averaged signal power for each condition, for standard, deviants, and their difference (deviant-minus-standard) in both groups and across seven frequency bands. We demonstrate the signal power for the whole set of trials and for the selection of a subset of trials.

**Figure 8:**
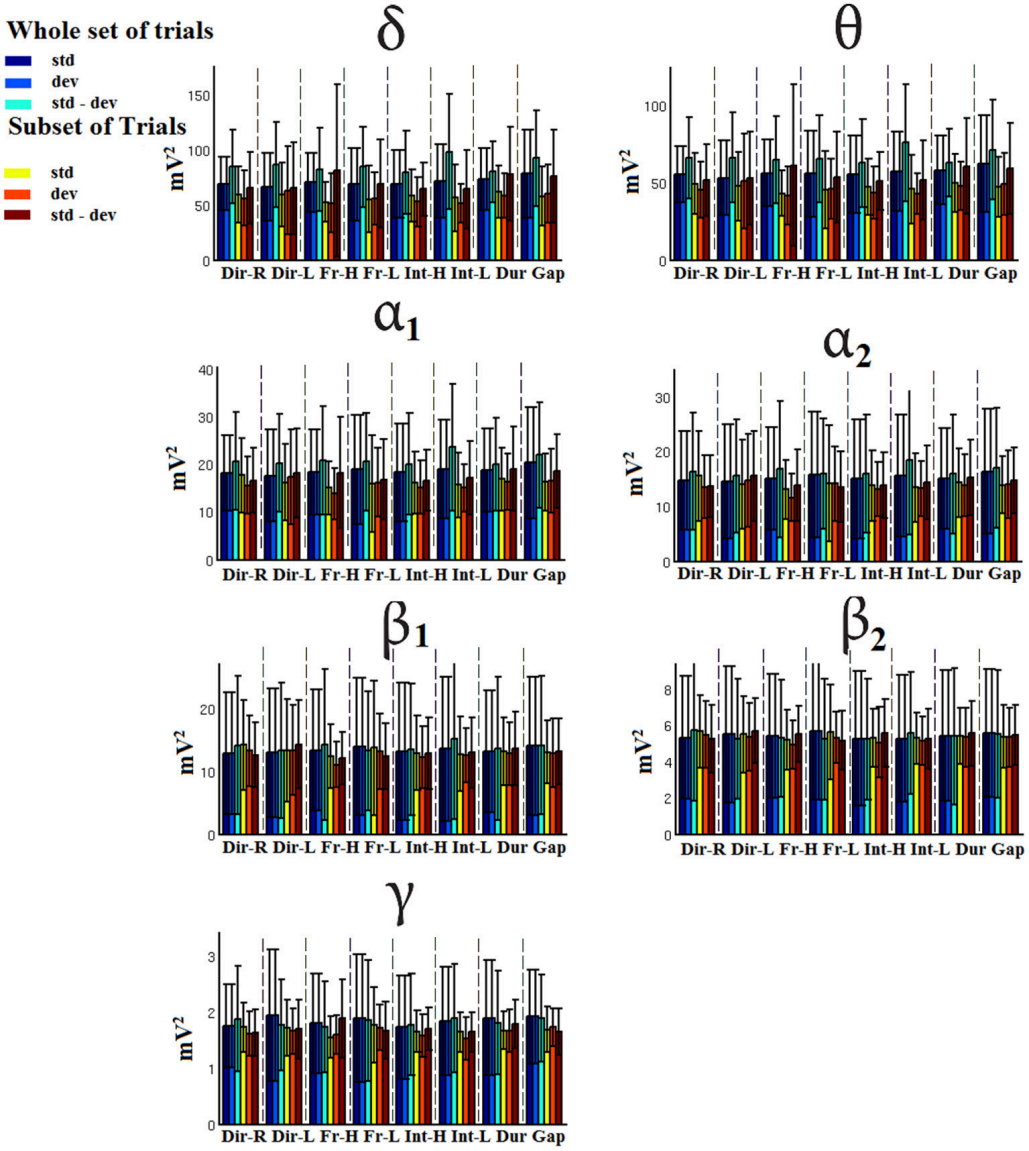
FCZ-Group-averaged signal power for each condition, for standard, deviants, and their difference (deviant-minus-standard) in both groups and across seven frequency bands. We demonstrate the signal power for the whole set of trials and for the selection of a subset of trials.

**Figure 9:**
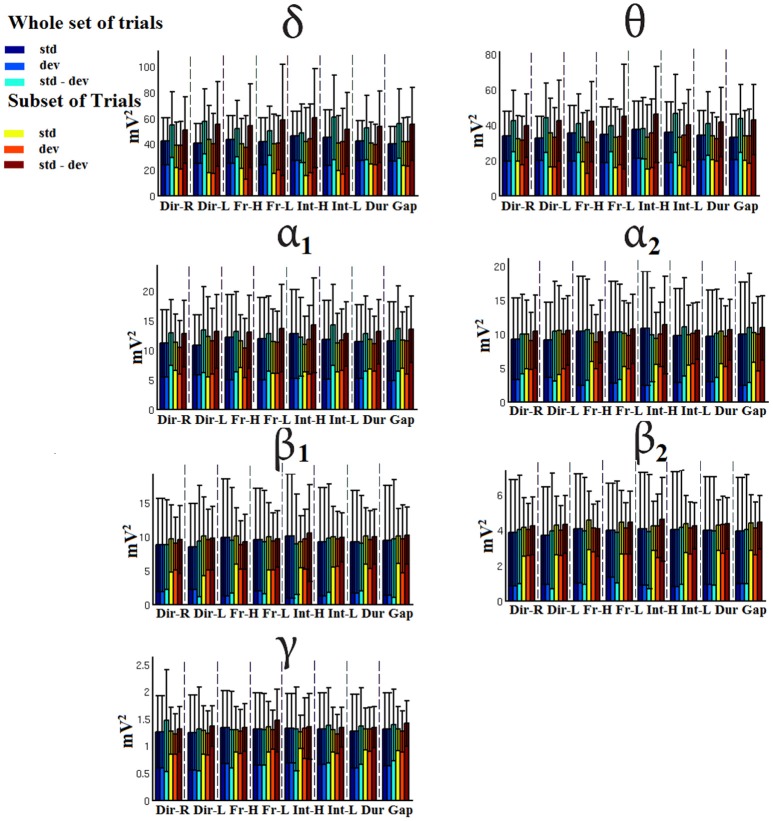
CZ-Group-averaged signal power for each condition, for standard, deviants, and their difference (deviant-minus-standard) in both groups and across seven frequency bands. We demonstrate the signal power for the whole set of trials and for the selection of a subset of trials.

Figures 7–9 demonstrated the group mean signal power for standard, deviant, and deviant – standard for each condition of the MMN experimental protocol and for the three EEG sensors. We estimated the signal power for every MMN feature for standard, deviant, and deviant – standard and for FZ (Figure 7), FCZ (Figure 8), and CZ (Figure 9) EEG sensors. In Figures 7–9, we demonstrated the signal power for each condition and std, dev, and std-dev for the whole set of trials and also for the selection of a subset of trials. CV of signal power was higher for the subset of trials compared to the whole set of trials.

### Variability of single-trials via global efficiency estimations

To assess the variability of STs, we estimated the global efficiency (GE) on a network level based on the subgraph defined by the nodes of GG linked to the extracted prototypical single-trials (red nodes in Figures 4, 5, 6E,G). We constructed the weighted graph by estimating the Euclidean distance between every pair of hub areas. Then, we normalized by the maximum value and we inversed the normalized weights in order to express functionality. This practically means that the higher the distance between two 2D points, the less efficient the communication. In a global level, the higher the GE the more efficient is the communication of the nodes within the network which further means that the nodes are closer in 2D points. So higher values of GE can be linked directly to less variability.

Table 8 tabulates the group-averaged GE for each condition in the three midline located EEG sensors for standard stimuli.

**Table 8:** Group-averaged GE for each condition across the three selected EEG sensors for (deviant-minus-standard) stimuli.

	**Dir-R**	**Dir-L**	**Freq-Hi**	**Freq-Low**	**Int-Hi**	**Int-Low**	**Duration**	**Gap**
FZ	0.37 ± 0.05	0.39 ± 0.07	0.41 ± 0.04	0.36 ± 0.04	0.42 ± 0.03	0.38 ± 0.06	0.36 ± 0.05	0.37 ± 0.04
	(7)	(5.6)	(10.25)	(9)	(14)	(6.3)	(7.2)	(9.25)
FCZ	0.34 ± 0.06	0.39 ± 0.04	0.37 ± 0.04	0.35 ± 0.04	0.37 ± 0.03	0.34 ± 0.03	0.38 ± 0.06	0.39 ± 0.06
	(5.6)	(9.8)	(9.25)	(8.75)	(12.3)	(11, 3)	(6.33)	(6.5)
CZ	0.39 ± 0.03	0.37 ± 0.04	0.36 ± 0.04	0.38 ± 0.04	0.38 ± 0.07	0.36 ± 0.04	0.38 ± 0.04	0.38 ± 0.06
	(13)	(9.25)	(9)	(9.5)	(5.4)	(9)	(9.5)	(6.33)

*Within the brackets we present the coefficient of variation (CV)*.

### Influence of EEG reference system to amplitude and latency estimation

We compared average to the REST reference system in terms of amplitude and latency estimation across EEG sensor locations and in standard, deviant, and standard-deviant stimuli. Comparing Tables 2–4 vs. Tables 9–11 for amplitude and Tables 5–7 vs. Tables 12–14 for latency, we revealed alterations of the group mean amplitude and latency. In both cases, the CV was too high while in some cases especially in the amplitude of deviant – standard, the CV was higher for the REST reference. For more detailed results, see section 1 in Supplementary Material.

**Table 9:** FZ EEG sensor: Group-averaged amplitude for each condition and for standard, deviants, and their difference (deviant-minus-standard).

	**Dir-R**	**Dir-L**	**Freq-Hi**	**Freq-Low**	**Int-Hi**	**Int-Low**	**Duration**	**Gap**
Std	4.3 ± 0.9(4.7)	4.3 ± 1.2(3.5)	4.0 ± 1.2(3.3)	4.6 ± 1.3(3.5)	4.7 ± 0.7(6.7)	4.5 ± 1.6(2.8)	2.9 ± 0.8(3.6)	3.4 ± 0.9(3.7)
Dev	−3.9 ± 1.2(3.2)	−6.7 ± 1.3(5.1)	−4.1 ± 1.2(3.4)	−4.5 ± 1.3(3.5)	−4.9 ± 0.9(5.4)	−4.8 ± 1.1(4.3)	−2.8 ± 0.5(5.6)	−2.9 ± 0.5(5.8)
Dev-Std	−5.1 ± 0.9(5.6)	−6.3 ± 0.9(7)	−5.1 ± 1.2(4.2)	−5.7 ± 1.1(5.1)	−4.6 ± 0.7(6.5)	−4.7 ± 1.2(3.9)	−3.7 ± 0.6(6.1)	−3.6 ± 0.5(7.2)

*Within the brackets we report the coefficient of variation (CV)*.

**Table 10:** FCZ EEG sensor: Group-averaged amplitude for each condition and for standard, deviants, and their difference (deviant-minus-standard).

	**Dir-R**	**Dir-L**	**Freq-Hi**	**Freq-Low**	**Int-Hi**	**Int-Low**	**Duration**	**Gap**
Std	3.7 ± 1.4(2.6)	3.7 ± 1.0(3.7)	3.8 ± 0.9(4.2)	3.8 ± 0.9(4.2)	3.7 ± 0.9(4.1)	3.7 ± 1.2(3.1)	2.7 ± 0.6(4.5)	2.5 ± 0.5(5)
Dev	−3.4 ± 1.3(2.6)	−3.8 ± 1.1(3.4)	−3.7 ± 1.0(3.7)	−3.9 ± 1.1(3.5)	−2.8 ± 0.8(3.5)	−3.5 ± 1.1(3.1)	−2.8 ± 0.5(5.8)	−2.8 ± 0.6(4.6)
Dev-Std	−4.6 ± 1.2(3.8)	−3.9 ± 0.8(4.8)	−4.2 ± 0.9(4.6)	−4.2 ± 1.2(3.5)	−4.3 ± 0.9(4.7)	−4.5 ± 1.1(4.1)	−3.5 ± 0.7(5)	−3.5 ± 0.7(5)

*Within the brackets we report the coefficient of variation (CV)*.

**Table 11:** CZ EEG sensor: Group-averaged amplitude for each condition and for standard, deviants, and their difference (deviant-minus-standard).

	**Dir-R**	**Dir-L**	**Freq-Hi**	**Freq-Low**	**Int-Hi**	**Int-Low**	**Duration**	**Gap**
Std	3.7 ± 1.1(3.3)	2.6 ± 0.9(2.8)	2.8 ± 0.8(3.5)	2.7 ± 0.8(3.3)	2.8 ± 0.9(3.1)	2.3 ± 0.8(2.8)	2.3 ± 0.5(4.6)	2.6 ± 0.4(6)
Dev	−2.5 ± 0.9(2.7)	−2.7 ± 1.0(2.7)	−2.7 ± 0.7(3.8)	−2.8 ± 0.9(3.1)	−2.9 ± 1.1(2.6)	−2.5 ± 0.9(2.7)	−2.6 ± 0.5(5.2)	−2.7 ± 0.5(5.4)
Dev-Std	−3.8 ± 1.1(3.4)	−3.7 ± 0.9(4.1)	−3.5 ± 0.8(4.3)	−3.7 ± 1.1(3.3)	−3.9 ± 0.8(4.8)	−2.9 ± 0.9(3.2)	−2.8 ± 0.7(4)	−3.3 ± 0.5(6.6)

*Within the brackets we reported the coefficient of variation (CV)*.

**Table 12:** FZ EEG sensor: Group-averaged latency for each condition and for standard, deviants, and their difference (deviant-minus-standard).

	**Dir-R**	**Dir-L**	**Freq-Hi**	**Freq-Low**	**Int-Hi**	**Int-Low**	**Duration**	**Gap**
Std	0.17 ± 0.03(5.6)	0.18 ± 0.03(6)	0.18 ± 0.02(9)	0.18 ± 0.03(6)	0.18 ± 0.03(6)	0.17 ± 0.03(5.6)	0.18 ± 0.03(6)	0.17 ± 0.03(5.6)
Dev	0.18 ± 0.03(6)	0.17 ± 0.03(5.6)	0.16 ± 0.03(6)	0.17 ± 0.03(5.6)	0.17 ± 0.02(8.5)	0.16 ± 0.03(5.3)	0.17 ± 0.03(5.6)	0.16 ± 0.03(5.3)
Dev-Std	0.16 ± 0.02(8)	0.15 ± 0.02(7.5)	0.15 ± 0.02(7.5)	0.15 ± 0.02(7.5)	0.16 ± 0.03(5.3)	0.15 ± 0.03(5)	0.16 ± 0.02(8)	0.16 ± 0.02(8)

*Within the brackets we reported the coefficient of variation (CV)*.

**Table 13:** FCZ EEG sensor: Group-averaged latency for each condition and for standard, deviants, and their difference (deviant-minus-standard).

	**Dir-R**	**Dir-L**	**Freq-Hi**	**Freq-Low**	**Int-Hi**	**Int-Low**	**Duration**	**Gap**
Std	0.18 ± 0.03(6)	0.17 ± 0.03(5.6)	0.18 ± 0.03(6)	0.17 ± 0.03(5.6)	0.17 ± 0.03(5.6)	0.17 ± 0.03(5.6)	0.17 ± 0.03(5.6)	0.18 ± 0.03(6)
Dev	0.17 ± 0.02(8.5)	0.18 ± 0.03(6)	0.17 ± 0.03(5.6)	0.15 ± 0.02(7.5)	0.18 ± 0.03(6)	0.17 ± 0.03(5.6)	0.19 ± 0.03(6.3)	0.19 ± 0.03(5.6)
Dev-Std	0.17 ± 0.02(8.5)	0.18 ± 0.02(9)	0.18 ± 0.03(6)	0.18 ± 0.03(6)	0.17 ± 0.03(5.6)	0.17 ± 0.02(8.5)	0.17 ± 0.03(5.6)	0.19 ± 0.02(8.5)

*Within the brackets we reported the coefficient of variation (CV)*.

**Table 14:** CZ EEG sensor: Group-averaged latency for each condition and for standard, deviants, and their difference (deviant-minus-standard).

	**Dir-R**	**Dir-L**	**Freq-Hi**	**Freq-Low**	**Int-Hi**	**Int-Low**	**Duration**	**Gap**
Std	0.17 ± 0.02(8.5)	0.18 ± 0.03(6)	0.18 ± 0.03(6)	0.17 ± 0.03(5.6)	0.18 ± 0.03(6)	0.17 ± 0.03(5.6)	0.18 ± 0.03(6)	0.17 ± 0.03(5.6)
Dev	0.18 ± 0.02(8.5)	0.19 ± 0.03(6.3)	0.18 ± 0.02(6)	0.15 ± 0.02(7.5)	0.17 ± 0.03(5.6)	0.18 ± 0.03(6)	0.19 ± 0.03(6.3)	0.18 ± 0.03(6)
Dev-Std	0.16 ± 0.02(8)	0.17 ± 0.02(8.5)	0.17 ± 0.03(5.6)	0.16 ± 0.03(5.6)	0.16 ± 0.03(5.6)	0.18 ± 0.02(9)	0.18 ± 0.03(6)	0.19 ± 0.02(9.5)

*Within the brackets we report the coefficient of variation (CV)*.

Tables 12–14 demonstrated the group mean latencies for standard, deviant, and deviant – standard for each condition of the MMN experimental protocol and for the three EEG sensors. We estimated the CV across the cohort for every MMN feature for standard, deviant, and deviant – standard and for FZ (Table 5), FCZ (Table 6), and CZ (Table 7) EEG sensors. It is clear that CV of the latency was higher for the FZ EEG sensor.

### Comparison with alternative mining algorithms

We compared our methodology with PCA and multi-linear regressor analysis (Hu et al., 2011). The first one proposed a multiple linear regression (MLR) and multiple linear regression with dispersion term (MLRd) to estimate the single-trial latency and amplitude of ERP peaks. Regressors (an average and its temporal derivative) for each ERP peak are calculated from the average ERP waveform within a given post-stimulus interval (in this case, 0–0.3 s) for each subject. These regressors are then applied against each ST within the same post-stimulus interval and used to model each single-trial ERP peak. In MLRd, variability matrices that capture the variations of latency and morphology of each ERP peak are generated by simultaneously shifting and compressing the average ERP waveform (step 1). These variability matrices, whose order of trials (with the latency shifted and the morphology varied simultaneously) is of no importance, are fed to a PCA (step 2). The resulting three main principal components (PCs) are used to define three regressors for each peak within a given post-stimulus interval (in this case, 0–0.5 s; step 3). These regressors are then applied against each ST within the same post-stimulus interval and used to model each single-trial ERP peak (step 4). The methodology is explained in details in Hu et al. (2011).

Since the original methodology focused on the estimation of amplitude-latency per single-trial, we grand-averaged the single-trials after first applying the regressors.

The second one is PCA where we kept the first PCs that explained more than 95% of the variance of single-trials.

**Figure 10:**
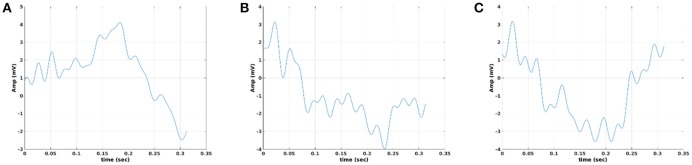
Grand-averaged response for DIR-L from subject 1 using multi-linear regressor analysis and average reference system (FZ-Sensor). **(A)** Standard stimulus, **(B)** Deviant stimulus, **(C)** Deviant-minus-standard stimulus.

**Figure 11:**
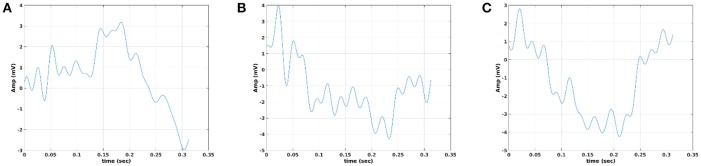
Grand-averaged response for DIR-L from subject 1 using multi-linear regressor analysis and REST reference system (FZ-Sensor). **(A)** Standard stimulus, **(B)** Deviant stimulus, **(C)** Deviant-minus-standard stimulus.

In Figure 10, we illustrate the resulting grand-averaged time series from subject 1 and stimulus DIR-L for standard, deviant, and deviant-minus-standard using the multi-linear regressor algorithm and the average reference system. Complementary, Figure 11 demonstrates the effect of REST reference on the grand-averaged time series illustrated in **Figure 16**. Both grand-averaged time series were extracted from FZ EEG sensor.

**Figure 12:**
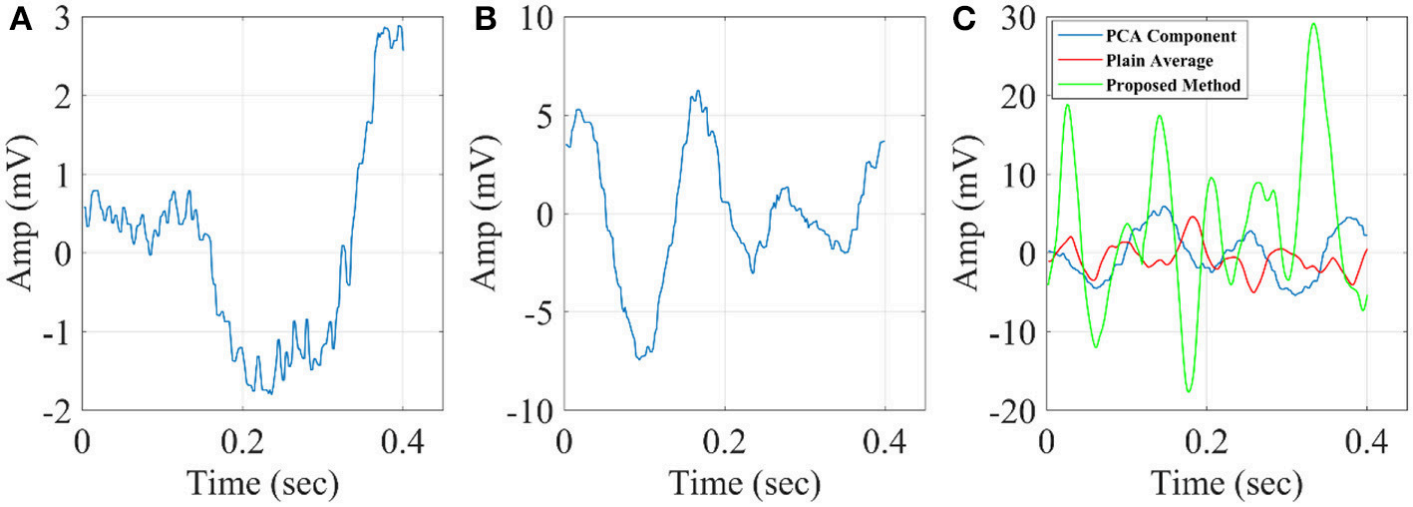
Grand-averaged response for DIR-L from subject 1 using PCA analysis and average reference system (FZ-Sensor). **(A)** Standard stimulus, **(B)** Deviant stimulus, **(C)** Deviant-minus-standard stimulus. We plotted the characteristic time series derived from PCA analysis, the plain average from the whole set of time series and the time series derived from the proposed method.

**Figure 13:**
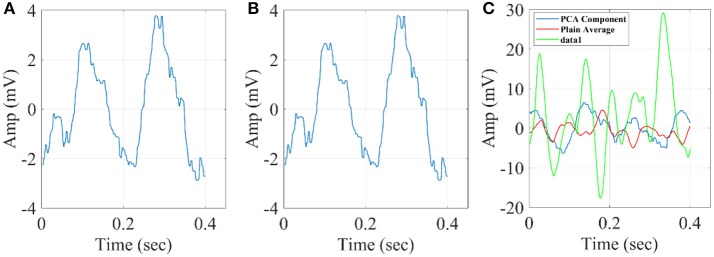
Grand-averaged response for DIR-L from subject 1 using PCA analysis and REST reference system (FZ-Sensor). **(A)** Standard stimulus, **(B)** Deviant stimulus, **(C)** Deviant-minus-standard stimulus. We plotted the characteristic time series derived from PCA analysis, the plain average from the whole set of time series and the time series derived from the proposed method.

In Figure 12, we illustrate the resulting grand-averaged time series from subject 1 and stimulus DIR-L for standard-deviant and deviant-minus-standard using PCA algorithm and the average reference system. On this exemplar, we demonstrate the 4th and 5th PC per case. Complementary, Figure 13 demonstrates the effect of REST reference on the grand-averaged time series illustrated in Figure 12. Both grand-averaged time series were extracted from FZ EEG sensor. We adopted the same stimulus, sensor location, and subject with multi-linear regressor analysis for comparison purposes between multi-linear regressor analysis and PCA. For further details, see section 2 in Supplementary Material.

**Figure 14:**
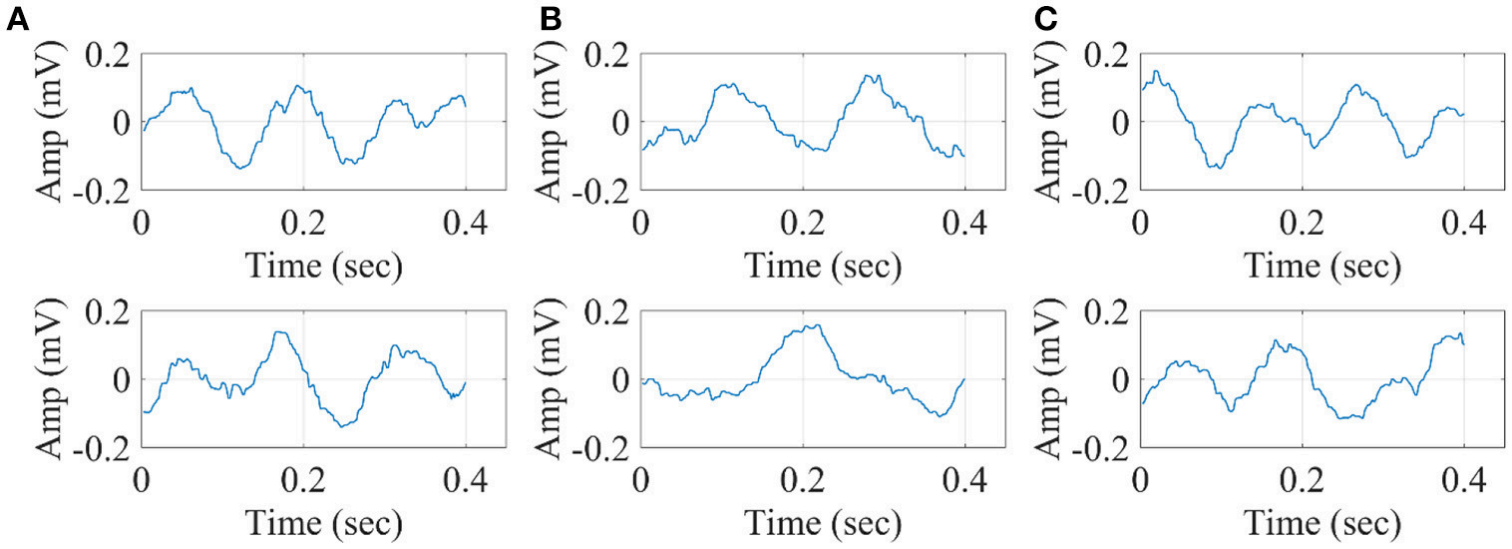
Grand-averaged of the 4th and 5th singular-values for DIR-L from subject 1 using SVD analysis and average reference system (FZ-Sensor). **(A)** Standard stimulus, **(B)** Deviant stimulus, **(C)** Deviant-minus-standard stimulus.

**Figure 15:**
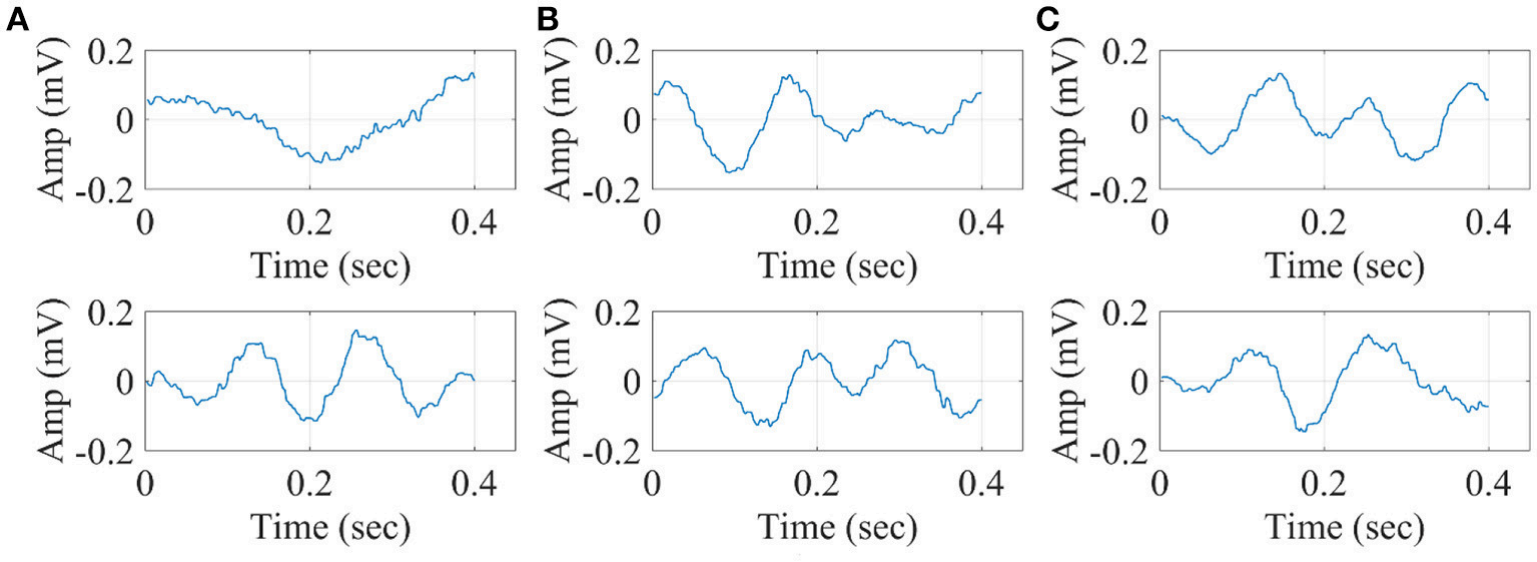
Grand-averaged of the 4th and 5th singular values for DIR-L from subject 1 using PCA analysis and REST reference system (FZ-Sensor). **(A)** Standard stimulus, **(B)** Deviant stimulus, **(C)** Deviant-minus-standard stimulus.

In Figure 14, we illustrate the resulting grand-averaged 4th and 5th singular time series from subject 1 and stimulus DIR-L for standard, deviant, and deviant-minus-standard using SVD algorithm and the average reference system. Complementary, Figure 15 demonstrates the effect of REST reference on the grand-averaged first two right singular time series illustrated in Figure 14. Both grand-averaged time series were extracted from FZ EEG sensor. We adopted the same stimulus, sensor location and subject with multi-linear regressor analysis for comparison purposes between multi-linear regressor analysis and PCA.

The three algorithms independently of the EEG reference system failed to detect an accurate amplitude and latency. The main reason is that both algorithms are sensitive to the grand-averaged response which in many cases like in MMN experimental paradigm are too noisy to get astable waveform that can be used as representative time series of brain response.

The proposed data mining scheme worked better compared to the three comparable techniques and also it is a parameter free method that can easily be used in any experimental multi-trial paradigm.

### Simulations

**Figure 16:**
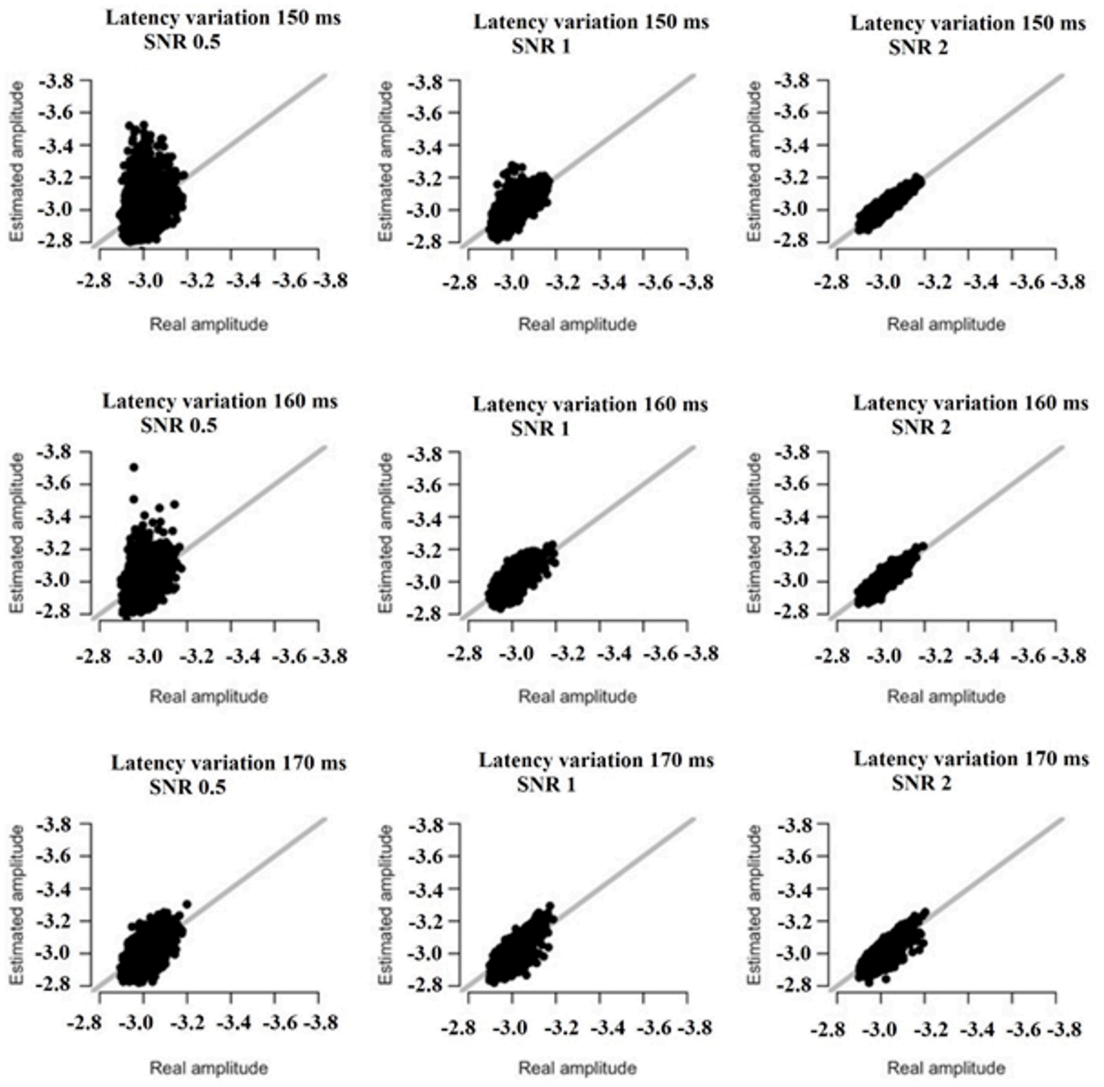
The simulation based on recordings derived from the FZ sensor in the DIR-L condition and for deviant-minus-standard stimulus. Real vs. estimated amplitude estimates for different amounts of SNR and latency variation.

**Figure 17:**
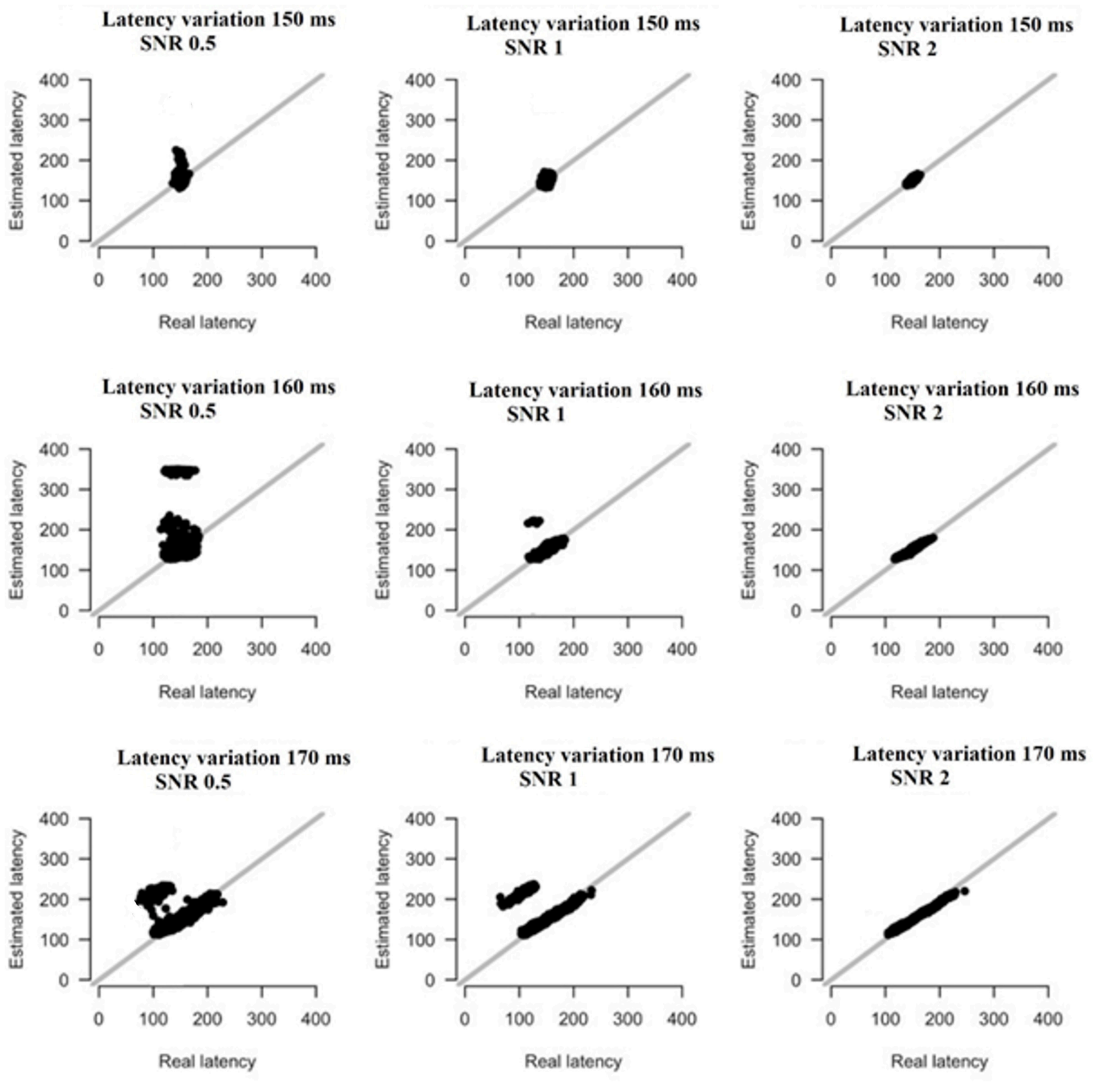
The simulation based on recordings derived from the FZ sensor in the DIR-L condition and for deviant-minus-standard stimulus. Real vs. estimated latency estimates for different amounts of SNR and latency variation.

Based on the results derived from the simulations, we revealed that both amplitude and latency are within acceptable limits. Simulations have shown that estimates of amplitude and latency are within acceptable limits (Figures 16, 17). Only if SNR is low and latency variation is low, estimates become unreliable. Figures 16, 17 illustrate the simulation based on recordings derived from the FZ sensor at DIR-L condition and for deviant-standard stimulus using the two simulated scenarios.

Finally, only in the case that both SNR and latency variation are low, estimates become unreliable (see Figures 16, 17).

## Discussion

The proposed methodology can reliably sample the representative single-trials in order to simultaneously express their variability and also to reconstruct a grand-average with high SNR. A high SNR was detected across subjects, conditions and recording EEG locations which secure a reliable estimation of the amplitude and latency of the characteristic peak elicited from the whole set of MMN features. The whole approach relies on graph theory by constructing a distance network from the 2D projected STs in a common feature space. The construction of the distance matrix based on members/nodes of proximity graph, called GG. Then, we detected from GG, the hubs nodes/single-trials using the degree *k* of each node as an input. These hubs can clearly describe the variability of single-trials and also reconstruct a waveform with a high SNR which clearly demonstrates a characteristic peak. We presented also how the final reconstructed waveform changed due to different types of filters and the related order. To get the best combination of filter type, order and selection of degree k directly linked to the selected hubs, we employed SNR as a true objective function. Our results can be summarized below:

- FIR filter with eegfilt function of order 2 and degree = 4 demonstrates the highest SNR across conditions and subjects
- The reliability of amplitude and latency was higher for FZ EEG sensor compared to FCZ and CZ based on CV
- CV of signal power was higher for the subset of trials compared to the whole set of trials.
- Amplitude and latency are sensitive to EEG reference system
- REST reference system improved the CV of amplitude in the deviant-minus-standard stimuli
- Similar amplitude/latency estimations were revealed with the 85–95% of the total amount of single-trials
- PCA, SVD, and multi-linear regressor manipulation of single-trials failed to retrieve a robust waveform, latency, and amplitude estimation.
- Amplitude and latency estimations with the proposed method are reliable unless SNR and latency variation is too low.

There are several extensions that can be applied to the present methodology in specific steps of the analytic algorithm. First of all, we demonstrated the effect of different filtering schemes where alternative adaptive filters can be used (Mandic and Goh, 2009). One can select different metrics to estimate the pair-wise correlation between single-trials. Complementary, one can use different members of proximity graphs like minimal spanning tree alone or in combination with voronoi testellation (Laskaris and Ioannides, 2001; Laskaris et al., 2004) and RNG. Here, we tested both of them but we revealed best results with GG. Additionally, it would be very interesting to apply source-localization algorithms on the representative single-trials to connect single-trials with sources and the timing of related activity (Laskaris and Ioannides, 2001; Laskaris et al., 2003). For example, one can localize the early segment of activity and the later one, in order to demonstrate the early activation of auditory cortex and the later activation in frontal lobe (Rinne et al., 2000).

Regarding the adopted experimental paradigm to demonstrate this methodology, the MMN mechanism consists of an auditory-based frontal lobe network. After the pre-processing of the content of MMN by the targeted sensory system here the auditory cortex, frontal areas are activated playing a significant role in the elicitation of a reflex (Näätänen and Michie, 1979). The MMN generators come from temporal and frontal lobes and the related activity is captured mainly by fronto-central EEG sensors (FZ, FCZ, CZ) and also from temporal electrodes (T3, T4) (Rinne et al., 2000). Two studies aimed to reveal, both with EEG and MEG recordings, the origin of the elicited activity linked to MMN. Dipole modeling techniques applied to MMN (Scherg et al., 1989) and its magnetic counterpart (MMNm) (Hari et al., 1984) were found to have generators in the auditory cortex and in the temporal lobes. Complementary, the analysis of scalp-potential distribution revealed a right-hemispheric MMN source, which mainly was located over the frontal lobe (Giard et al., 1990; Deouell et al., 1998). A more recent paper using a simultaneous EEG-MEG recording set up, source-localized both EEG and MEG activity in an auditory MMN (Rinne et al., 2000). They validated the hypothesis that frontal MMN generators are activated later than generator in the auditory cortex. For a review of MMN generators in both healthy and disease groups and various settings, an interested reader can refer to a detailed review (Garrido et al., 2009).

The MMN is an ERP elicited by the occurrence of a rare event (deviance) in a regular acoustic environment, and is assumed to reflect a pre-attentive mechanism for change detection. Cortical generators of MMN are located in the superior temporal planes bilaterally which are responsible for the sensory memory part of change detection and frontal lobe sources responsible for triggering an attention shift upon change detection (for a review see Deouell, 2007). These bilateral temporal-frontal generators of MMN can be better detected with EEG compared to MEG while the combination of both modalities was suggested (Hämäläinen et al., 1993). Apart from bilateral auditory-cortex activation which underlines a pre-perceptual change detection with a short time-delay (Rinne et al., 2000), a predominant right hemispheric frontal process could be detected linked to involuntary attention switch to auditory change (Rinne et al., 2005, 2006). The dominant hemisphere of the MMN response due to acoustic changes is the right hemisphere (Levänen et al., 1996). For that reason, it is important in a next study to further explore amplitude-latency estimations also in bilateral frontal electrode sites complementary to EEG sensors located in the midline.

In the present study, we focused on the presentation of a data-driven methodology for a proper analysis of single-trials. We demonstrated high reliability in amplitude, latency, variability, and signal power for the whole cohort of young adults. Additionally, the majority of the fronto-central EEG channels should be studied to uncover any significant asymmetries of the brain activity between the two groups. Complementary, the main focus of this study was to enhance the reliability of the proposed methodology to reveal high SNR grand-averaged trials in various MMN conditions and reliable estimates of amplitude, latency, and variability in a healthy group. The REST reference system improved the CV of amplitude in the deviant-minus-standard stimuli while PCA, SVD, and multi-linear regressor manipulation of single-trials failed to retrieve a robust waveform, latency, and amplitude estimation. The proposed data-driven scheme worked better compared to the three well-known comparable methodologies. Moreover, it is parameter free method that can easily be adjusted to any multi-trial experimental paradigm using EEG-MEG recordings at both sensor and source levels. Finally, amplitude and latency estimations with the proposed method are reliable unless SNR and latency variation is too low.

The whole methodology will be valuable for neuroscientists particularly interest in defining a reliable biomarker based on ERP studies in various cognitive states (Picton et al., 2000; Espeseth et al., 2009; Horvath et al., 2018) and also in disease brain states such as the Alzheimer's Disease (Tsolaki et al., 2017).

## Conclusions

We presented a fast, reliable, and data-driven methodology for simultaneously data-mining single-trials and amplitude-latency estimation. The method relies on graph and network analysis as appropriate tools of geometrical data analysis and vectorial pattern analytic tools of single-trials. We demonstrated the effect of filtering settings on the grand-averaged trial and the related amplitude-latency estimates. Additionally, the whole methodology was presented in an auditory EEG MMN task with the aim to detect reliable amplitude, latency, and signal power derived from the appropriate preselection of single-trials. Based on the data-driven approach of the current methodology, the whole analysis could be of high value for various evoked/event-related potentials in various neuroimaging studies including EEG, MEG, and fMRI.

## Author contributions

Conception of the research, methods and design, data analysis, and drafting the manuscript: SD; Critical revision of the manuscript: LB, LE, DL, KS; All authors read and approved the final version of the manuscript.

### Conflict of interest statement

The authors declare that the research was conducted in the absence of any commercial or financial relationships that could be construed as a potential conflict of interest.
