# Supplementary material for: A Novel, Fast, Reliable, and Data-Driven Method for Simultaneous Single-Trial Mining and Amplitude—Latency Estimation Based on Proximity Graphs and Network Analysis
Source: Front Neuroinform. 2018 Nov 19;12:59. doi: 10.3389/fninf.2018.00059 (PMC6252329; doi:10.3389/fninf.2018.00059)
Supplement: Supplementary file 1 [file Data_Sheet_1.docx]

**Supplementary Material**

**A Novel, Fast, Reliable and Data-Driven Method for Simultaneous Single-Trial Mining and Amplitude - Latency Estimation Based on Proximity Graphs and Network Analysis**

**Dimitriadis SI^1-6*,^Brindley L^7^, Evans L^1,4^, Linden DE^1,3,4,5,6^, Singh K^1,4^**

1 Cardiff University Brain Research Imaging Centre, School of Psychology, Cardiff University, Cardiff, United Kingdom,

2 Neuroinformatics Group, Cardiff University Brain Research Imaging Centre, School of Psychology, Cardiff University, Cardiff,

United Kingdom, 3 Division of Psychological Medicine and Clinical Neurosciences, School of Medicine, Cardiff University,

Cardiff, United Kingdom, 4 School of Psychology, Cardiff University, Cardiff, United Kingdom, 5 Neuroscience and Mental

Health Research Institute, School of Medicine, Cardiff University, Cardiff, United Kingdom, 6MRC Centre for Neuropsychiatric

Genetics and Genomics, School of Medicine, Cardiff University, Cardiff, United Kingdom, 7 Department of Psychology, Cardiff

Metropolitan University, Cardiff, United Kingdom

***Corresponding author:**

Dr.Dimitriadis Stavros

Research Fellow

Institute of Psychological Medicine and Clinical Neurosciences, Cardiff University School of Medicine, Cardiff, United Kingdom

**Email**: [stidimitriadis@gmail.com](mailto:stidimitriadis@gmail.com) ; [DimitriadisS@cardiff.ac.uk](mailto:DimitriadisS@cardiff.ac.uk)

1. **The Influence of REST Infinity Reference on Amplitude and Latency Estimations**

**We applied REST re-reference on the scalp EEG recordings (Yao,2001 ; Yao et al., 2005) and we repeated our methodology. S.**Tables 1-3 demonstrated the group mean amplitude for standard, deviant and deviant – standard for each condition of the MMN experimental protocol and for the three EEG sensors. We estimated the coefficient of variation (CV) across the cohort for every MMN feature for standard, deviant and deviant – standard and for FZ (S.Table 1) , FCZ (S.Table 2) and CZ (S.Table 3) EEG sensors. It is obvious that CV of the amplitude was higher for FZ EEG sensor.

**S.Table 1. FZ EEG sensor:** Group-averaged amplitude for each condition and for standard,deviants and their difference (deviant - standard). Within the brackets, we reported the coefficient of variation (CV).

|  | **Dir-R** | **Dir-L** | **Freq-Hi** | **Freq-Low** | **Int-Hi** | **Int-Low** | **Duration** | **Gap** |
| --- | --- | --- | --- | --- | --- | --- | --- | --- |
| **Std** | 4.3 $\pm$ 0.9(4.7) | 4.3 $\pm$ 1.2(3.5) | 4.0 $\pm$ 1.2(3.3) | 4.6$\pm$ 1.3 (3.5) | 4.7 $\pm$ 0.7(6.7) | 4.5 $\pm$ 1.6(2.8) | 2.9 $\pm$ 0.8(3.6) | 3.4 $\pm$ 0.9(3.7) |
| **Dev** | -3.9 $\pm$ 1.2(3.2) | -6.7 $\pm$ 1.3 (5.1) | -4.1 $\pm$ 1.2 (3.4) | -4.5 $\pm$ 1.3 (3.5) | -4.9 $\pm$ 0.9(5.4) | -4.8 $\pm$ 1.1(4.3) | -2.8 $\pm$ 0.5(5.6) | -2.9 $\pm$ 0.5(5.8) |
| **Dev-Std** | -5.1 $\pm$ 0.9 (5.6) | -6.3 $\pm$ 0.9 (7) | -5.1 $\pm$ 1.2 (4.2) | -5.7 $\pm$ 1.1(5.1) | -4.6 $\pm$ 0.7(6.5) | -4.7 $\pm$ 1.2 (3.9) | -3.7 $\pm$ 0.6 (6.1) | -3.6 $\pm$ 0.5(7.2) |

**S.Table 2. FCZ EEG sensor:** Group-averaged amplitude for each condition and for standard,deviants and their difference (deviant - standard). Within the brackets, we reported the coefficient of variation (CV).

|  | **Dir-R** | **Dir-L** | **Freq-Hi** | **Freq-Low** | **Int-Hi** | **Int-Low** | **Duration** | **Gap** |
| --- | --- | --- | --- | --- | --- | --- | --- | --- |
| **Std** | 3.7 $\pm$ 1.4 (2.6) | 3.7 $\pm$ 1.0(3.7) | 3.8 $\pm$ 0.9(4.2) | 3.8$\pm$ 0.9 (4.2) | 3.7 $\pm$ 0.9(4.1) | 3.7 $\pm$ 1.2(3.1) | 2.7 $\pm$ 0.6(4.5) | 2.5 $\pm$ 0.5 (5) |
| **Dev** | -3.4 $\pm$ 1.3 (2.6) | -3.8 $\pm$ 1.1 (3.4) | -3.7 $\pm$ 1.0 (3.7) | -3.9 $\pm$ 1.1 (3.5) | -2.8 $\pm$ 0.8(3.5) | -3.5 $\pm$ 1.1(3.1) | -2.8 $\pm$ 0.5(5.8) | -2.8 $\pm$ 0.6 (4.6) |
| **Dev-Std** | -4.6 $\pm$ 1.2 (3.8) | -3.9 $\pm$ 0.8 (4.8) | -4.2 $\pm$ 0.9 (4.6) | -4.2 $\pm$ 1.2 (3.5) | -4.3 $\pm$ 0.9(4.7) | -4.5 $\pm$ 1.1 (4.1) | -3.5 $\pm$ 0.7 (5) | -3.5 $\pm$ 0.7 (5) |

**S.Table 3. CZ EEG sensor:** Group-averaged amplitude for each condition and for standard,deviants and their difference (deviant - standard). Within the brackets, we reported the coefficient of variation (CV).

|  | **Dir-R** | **Dir-L** | **Freq-Hi** | **Freq-Low** | **Int-Hi** | **Int-Low** | **Duration** | **Gap** |
| --- | --- | --- | --- | --- | --- | --- | --- | --- |
| **Std** | 3.7$\pm$ 1.1 (3.3) | 2.6 $\pm$ 0.9(2.8) | 2.8 $\pm$ 0.8 (3.5) | 2.7$\pm$ 0.8 (3.3) | 2.8 $\pm$ 0.9(3.1) | 2.3 $\pm$ 0.8 (2.8) | 2.3 $\pm$ 0.5(4.6) | 2.6 $\pm$ 0.4 (6) |
| **Dev** | -2.5 $\pm$ 0.9 (2.7) | -2.7 $\pm$ 1.0 (2.7) | -2.7 $\pm$ 0.7 (3.8) | -2.8 $\pm$ 0.9 (3.1) | -2.9 $\pm$ 1.1(2.6) | -2.5 $\pm$ 0.9(2.7) | -2.6 $\pm$ 0.5(5.2) | -2.7 $\pm$ 0.5 (5.4) |
| **Dev-Std** | -3.8 $\pm$ 1.1 (3.4) | -3.7 $\pm$ 0.9 (4.1) | -3.5 $\pm$ 0.8 (4.3) | -3.7 $\pm$ 1.1 (3.3) | -3.9 $\pm$ 0.8(4.8) | -2.9 $\pm$ 0.9 (3.2) | -2.8 $\pm$ 0.7 (4) | -3.3 $\pm$ 0.5 (6.6) |

S.Tables 4-6 demonstrated the group mean latencies for standard, deviant and deviant – standard for each condition of the MMN experimental protocol and for the three EEG sensors. We estimated the coefficient of variation (CV) across the cohort for every MMN feature for standard, deviant and deviant – standard and for FZ (Table 6) , FCZ (Table 7) and CZ (Table 8) EEG sensors. It is obvious that CV of the latency was higher for FZ EEG sensor.

**S.Table 4. FZ EEG sensor:** Group-averaged latency for each condition and for standard,deviants and their difference (deviant - standard). Within the brackets, we reported the coefficient of variation (CV).

|  | **Dir-R** | **Dir-L** | **Freq-Hi** | **Freq-Low** | **Int-Hi** | **Int-Low** | **Duration** | **Gap** |
| --- | --- | --- | --- | --- | --- | --- | --- | --- |
| **Std** | 0.17 $\pm$ 0.03(5.6) | 0.18 $\pm$ 0.03(6) | 0.18 $\pm$ 0.02 (9) | 0.18$\pm$ 0.03 (6) | 0.18$\pm$ 0.03(6) | 0.17 $\pm$ 0.03 (5.6) | 0.18 $\pm$ 0.03(6) | 0.17 $\pm$ 0.03 (5.6) |
| **Dev** | 0.18 $\pm$ 0.03 (6) | 0.17 $\pm$ 0.03(5.6) | 0.16 $\pm$ 0.03(6) | 0.17 $\pm$ 0.03(5.6) | 0.17 $\pm$ 0.02(8.5) | 0.16 $\pm$ 0.03(5.3) | 0.17 $\pm$ 0.03(5.6) | 0.16 $\pm$ 0.03(5.3) |
| **Dev-Std** | 0.16 $\pm$ 0.02 (8) | 0.15 $\pm$ 0.02 (7.5) | 0.15 $\pm$ 0.02 (7.5) | 0.15 $\pm$ 0.02(7.5) | 0.16 $\pm$ 0.03(5.3) | 0.15 $\pm$ 0.03 (5) | 0.16 $\pm$ 0.02 (8) | 0.16 $\pm$ 0.02 (8) |

**S.Table 5. FCZ EEG sensor:** Group-averaged latency for each condition and for standard,deviants and their difference (deviant - standard). Within the brackets, we reported the coefficient of variation (CV).

|  | **Dir-R** | **Dir-L** | **Freq-Hi** | **Freq-Low** | **Int-Hi** | **Int-Low** | **Duration** | **Gap** |
| --- | --- | --- | --- | --- | --- | --- | --- | --- |
| **Std** | 0.18 $\pm$ 0.03(6) | 0.17 $\pm$ 0.03(5.6) | 0.18 $\pm$ 0.03 (6) | 0.17$\pm$ 0.03 (5.6) | 0.17$\pm$ 0.03(5.6) | 0.17 $\pm$ 0.03 (5.6) | 0.17 $\pm$ 0.03(5.6) | 0.18 $\pm$ 0.03 (6) |
| **Dev** | 0.17 $\pm$ 0.02 (8.5) | 0.18 $\pm$ 0.03(6) | 0.17 $\pm$ 0.03 (5.6) | 0.15 $\pm$ 0.02 (7.5) | 0.18 $\pm$ 0.03(6) | 0.17 $\pm$ 0.03(5.6) | 0.19 $\pm$ 0.03(6.3) | 0.19 $\pm$ 0.03(5.6) |
| **Dev-Std** | 0.17 $\pm$ 0.02 (8.5) | 0.18 $\pm$ 0.02 (9) | 0.18 $\pm$ 0.03 (6) | 0.18 $\pm$ 0.03 (6) | 0.17 $\pm$ 0.03(5.6) | 0.17 $\pm$ 0.02 (8.5) | 0.17 $\pm$ 0.03 (5.6) | 0.19 $\pm$ 0.02 (8.5) |

**S.Table 6. CZ EEG sensor:** Group-averaged latency for each condition and for standard,deviants and their difference (deviant - standard). Within the brackets, we reported the coefficient of variation (CV).

|  | **Dir-R** | **Dir-L** | **Freq-Hi** | **Freq-Low** | **Int-Hi** | **Int-Low** | **Duration** | **Gap** |
| --- | --- | --- | --- | --- | --- | --- | --- | --- |
| **Std** | 0.17 $\pm$ 0.02 (8.5) | 0.18 $\pm$ 0.03 (6) | 0.18 $\pm$ 0.03 (6) | 0.17$\pm$ 0.03 (5.6) | 0.18$\pm$ 0.03(6) | 0.17 $\pm$ 0.03 (5.6) | 0.18 $\pm$ 0.03(6) | 0.17 $\pm$ 0.03 (5.6) |
| **Dev** | 0.18 $\pm$ 0.02 (8.5) | 0.19 $\pm$ 0.03 (6.3) | 0.18 $\pm$ 0.02 (6) | 0.15 $\pm$ 0.02 (7.5) | 0.17 $\pm$ 0.03(5.6) | 0.18 $\pm$ 0.03(6) | 0.19 $\pm$ 0.03(6.3) | 0.18 $\pm$ 0.03(6) |
| **Dev-Std** | 0.16 $\pm$ 0.02 (8) | 0.17 $\pm$ 0.02 (8.5) | 0.17 $\pm$ 0.03 (5.6) | 0.16 $\pm$ 0.03 (5.6) | 0.16 $\pm$ 0.03(5.6) | 0.18 $\pm$ 0.02 (9) | 0.18 $\pm$ 0.03 (6) | 0.19 $\pm$ 0.02 (9.5) |

1. **Comparison of Graph-based Single-Trial Mining Approach with other methods**

We adopted two algorithmic pipelines to compare the proposed method with existing ones. The first one proposed a multiple linear regression (MLR) and multiple linear regression with dispersion term (MLRd) to estimate the single-trial latency and amplitude of event-related potential (ERP) peaks. Regressors (an average and its temporal derivative) for each ERP peak are calculated from the average ERP waveform within a given post-stimulus interval (in this case, 0 to 0.3 s) for each subject. These regressors are then applied against each single trial within the same post-stimulus interval and used to model each single-trial ERP peak. In MLR d, variability matrices that capture the variations of latency and morphology of each ERP peak are generated by simultaneously shifting and compressing the average ERP waveform (step 1). These variability matrices, whose order of trials (with the latency shifted and the morphology varied simultaneously) is of no importance, are fed to a principal component analysis (PCA; step 2). The resulting 3 main principal components (PCs) are used to deﬁne 3 regressors for each peak within a given post-stimulus interval (in this case, 0 to 0.5 s; step 3). These regressors are then applied against each single trial within the same post-stimulus interval and used to model each single-trial ERP peak (step 4). The methodology is explained in details in Hu et al., 2011.

Since the original methodology focused on the estimation of amplitude-latency per single-trial, we grand-averaged the single-trials after first applying the regressors.

The second one is principal component analysis (PCA) where we kept the first PCs that explained more than 95% of the variance of single-trials.

In S.1, we illustrated the resulting grand-averaged time series from subject 1 and stimulus DIR-L for standard,deviant and deviant-minus-standard using the multi-linear regressor algorithm and the average reference system. Complementary, S.2 demonstrates the effect of REST reference on the grand-averaged time series illustrated in S.1. Both grand-averaged time series were extracted from FZ EEG sensor.


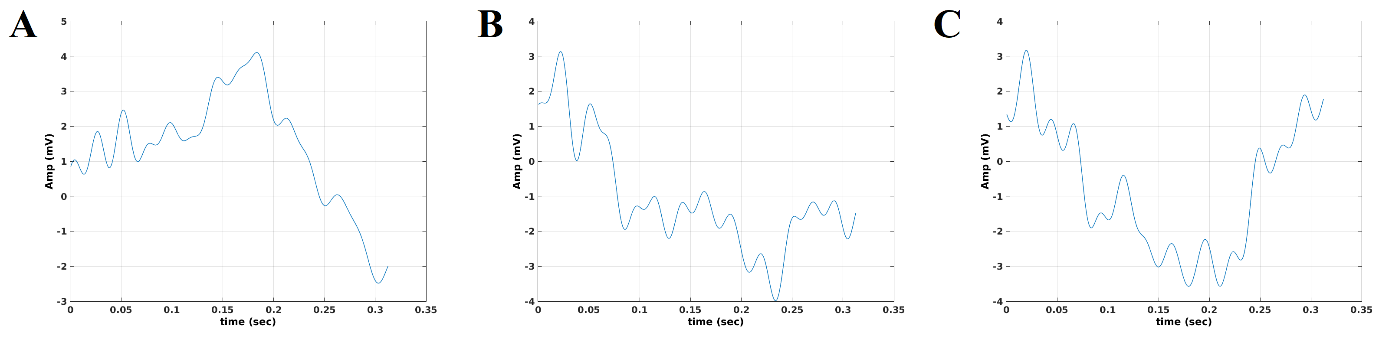


**S.1. Grand-averaged response for DIR-L from subject 1 using multi-linear regressor analysis and average reference system (FZ-Sensor).**

A.Standard stimulus

B.Deviant stimulus

C.Deviant-minus-standard stimulus


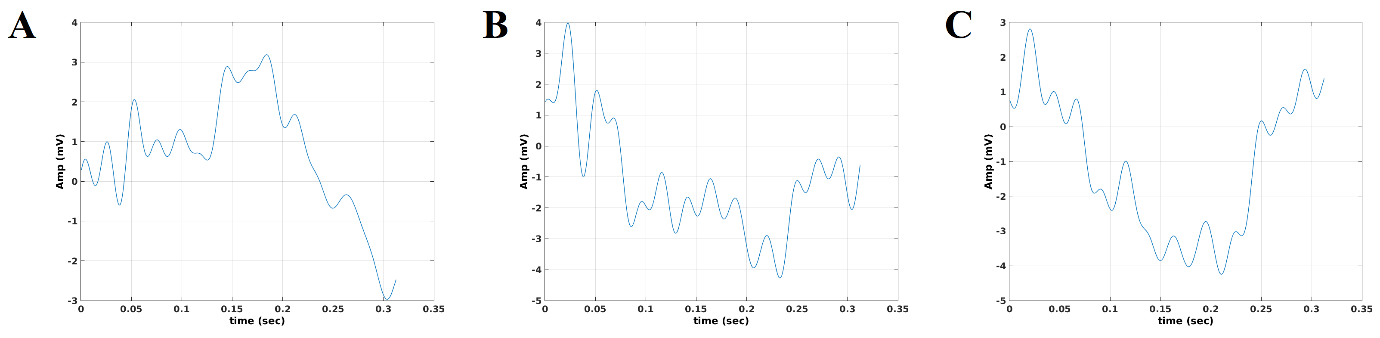


**S.2. Grand-averaged response for DIR-L from subject 1 using multi-linear regressor analysis and REST reference system (FZ-Sensor).**

A.Standard stimulus

B.Deviant stimulus

C.Deviant-minus-standard stimulus

In S.3, we illustrated the resulting grand-averaged time series from subject 1 and stimulus DIR-L for standard,deviant and deviant-minus-standard using PCA algorithm and the average reference system. On this exemplar, we kept the first PCs that kept more than 95% of the variance of single-trials. Complementary, S.4 demonstrates the effect of REST reference on the grand-averaged time series illustrated in S.1. Both grand-averaged time series were extracted from FZ EEG sensor. We adopted the same stimulus,sensor location and subject with multi-linear regressor analysis for comparison purposes between multi-linear regressor analysis and PCA.


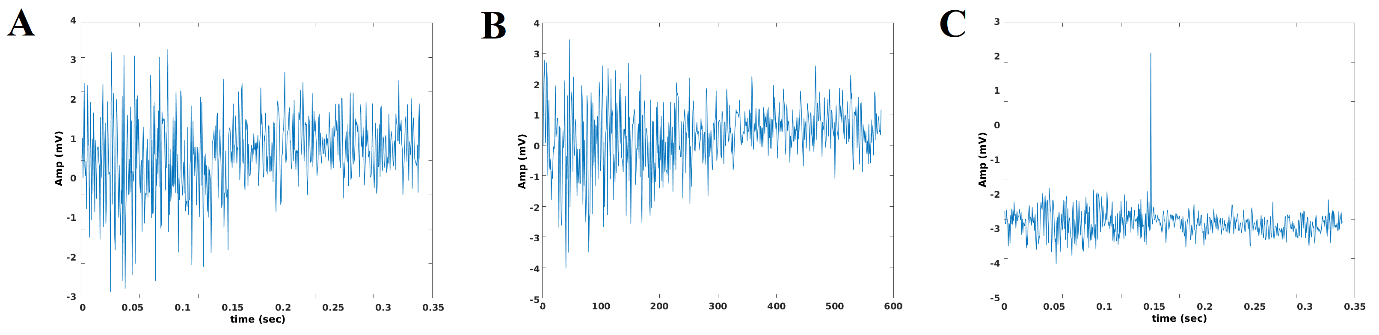


**S.1. Grand-averaged response for DIR-L from subject 1 using PCA analysis and average reference system (FZ-Sensor).**

A.Standard stimulus

B.Deviant stimulus

C.Deviant-minus-standard stimulus


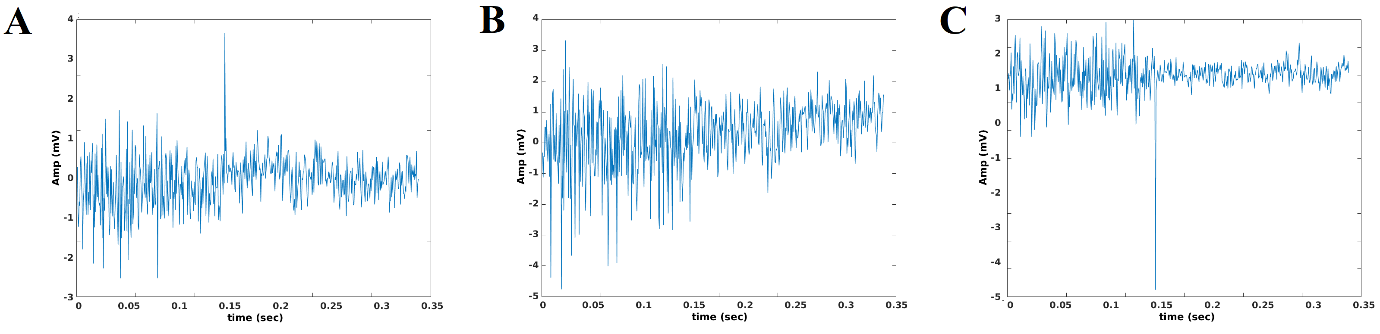


**S.4. Grand-averaged response for DIR-L from subject 1 using PCA analysis and REST reference system (FZ-Sensor).**

A.Standard stimulus

B.Deviant stimulus

C.Deviant-minus-standard stimulus

**References**

De Vico Fallani F, Maglione A, Babiloni F, Mattia D, Astolfi L, Vecchiato G, De Rinaldis A, Salinari S, Pachou E, Micheloyannis S. Cortical network analysis in patients affected by schizophrenia. Brain Topogr. 2010 Jun; 23(2):214-20.

Hu,L M. Liang, A. Mouraux, R. G. Wise, Y. Hu, and G. D. Iannetti (2011). Taking into account latency, amplitude, and morphology: improved estimation of single-trial ERPs by wavelet ﬁltering and multiple linear regression. J Neurophysiol 106: 3216–3229

Yao, D. (2001). A method to standardize a reference of scalp EEG recordings to a point at infinity. *Physiol. Meas.* 22, 693–711. doi: 10.1088/0967-3334/22/4/305

Yao, D., Wang, L., Oostenveld, R., Nielsen, K. D., Arendt-Nielsen, L., and Chen, A. C. (2005). A comparative study of different references for EEG spectral mapping: the issue of the neutral reference and the use of the infinity reference. *Physiol. Meas.* 26, 173–184. doi: 10.1088/0967-3334/26/3/003
